# Neuroimaging and Neuromonitoring Effects of Electro and Manual Acupuncture on the Central Nervous System: A Literature Review and Analysis

**DOI:** 10.1155/2015/641742

**Published:** 2015-08-03

**Authors:** Brigitte Elisabeth Scheffold, Ching-Liang Hsieh, Gerhard Litscher

**Affiliations:** ^1^Graduate Institute of Acupuncture Science, International Master Program, China Medical University, Taichung 40402, Taiwan; ^2^Graduate Institute of Integrated Medicine, China Medical University, Taichung 40402, Taiwan; ^3^China Medical University, Taichung 40402, Taiwan; ^4^Research Unit for Complementary and Integrative Laser Medicine, Research Unit of Biomedical Engineering in Anesthesia and Intensive Care Medicine, and TCM Research Center Graz, Medical University of Graz, 8036 Graz, Austria

## Abstract

The aim of this review is to provide an overview of the different effects of manual and electroacupuncture on the central nervous system in studies with different neuroimaging interventions. The Database PubMed was searched from 1/1/2000 to 1/6/2014 with restriction to human studies in English language. Data collection for functional magnetic resonance (fMRI) studies was restricted to the period from 1/1/2010 to 1/6/2014 due to a recently published review which included all published randomized and nonrandomized controlled clinical studies as well as observational studies with control groups, no blinding required. Only studies comparing manual or electroacupuncture with sham acupuncture were eligible. All participants were healthy adult men and women. A majority of 25 studies compared manual versus sham, a minority of 7 trials compared electro versus sham and only 1 study compared electro versus manual acupuncture. In 29 out of 33 studies verum acupuncture results were found to present either more or different modulation effects on neurological components measured by neuroimaging and neuromonitoring methods than sham acupuncture. Only four studies reported no effects of verum in comparison to sham acupuncture. Evaluation of the very heterogeneous results shows evidence that verum acupuncture elicits more modulation effects on neurological components than sham acupuncture.

## 1. Introduction

Acupuncture has been used as a traditional medical treatment in China for over 2000 years [1] and is now rapidly gaining popularity in the field of western complementary medicine [2].

In 2007, the World Health Organization (WHO) defined acupuncture as the insertion of needles into the human body for therapeutic purposes. However, treatment styles vary significantly in terms of stimulation (manual or electrical), manipulation (tonifying or draining), needling depth, and duration of needle retention. Likewise, different styles of acupuncture can elicit various needling sensations called deqi, which can be described as soreness, numbness, distension, heaviness, or electric shock sensation [3].

Depending on the style of application and the related deqi sensation, acupuncture evokes several complex somatosensory stimulations [4]. The following effects in the central nervous system might regulate homeostatic balance and modulate cognitive affective pain perception through a network of brain areas involved in sensory, autonomic, and cognitive/affect processing [5].

Even though many studies about neurophysiologic correlates have been done, the specific effects of acupuncture mechanisms on the central nervous system (CNS) still remain unclear. In the past decade an increasing number of studies used modern neuroimaging modalities like functional magnetic resonance imaging (fMRI), positron emission tomography (PET), and electroencephalography (EEG) for further investigation.

Using neuroimaging technologies, researchers are able to examine the acupuncture process in the brain noninvasively. Due to their good spatial resolution fMRI and PET are especially suitable for investigating the localization of active brain networks, whereas a comparatively better temporal resolution makes EEG and evoked potentials (EP) suitable for investigating the timing of activation [5].

This review paper presents a summary of current studies about neuroimaging technologies in acupuncture research. Data will be discussed regarding aspects of research methodology and the according challenges. For this purpose, the study outcomes will be compared in several subgroups.

The results shall provide an overview on neurophysiologic correlates of different acupuncture modalities in the brain.

## 2. Background

### 2.1. Neuroimaging Technologies

**Figure 1:**
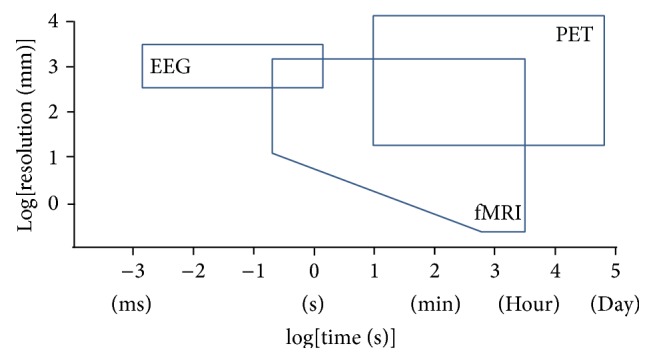
Spatial and temporal resolution of MRI, PET, and EEG (modified from [117]).

Figure 1 gives an overview of the temporal and spatial resolution of the different neuroimaging techniques this review is dedicated to.

#### 2.1.1. Functional Magnetic Resonance Imaging (fMRI)

fMRI measures the so-called BOLD (hemodynamic blood oxygenation level dependent) effect, which reflects the ratio between oxygenated and deoxygenated hemoglobin. This ratio represents the brain's neuronal activity and the resulting regional changes in metabolism and circulation. fMRI has a high spatial resolution (1–3 mm^3^) but limited temporal resolution as the hemodynamic response peaks 4-5 seconds after neuronal activity [6].

For research settings, it is very important to choose an adequate fMRI experimental design in order to enable suitable data analysis. The most common research designs are block or event related designs. Optimized designs for localizing brain activity usually apply the general linear model (GLM), the independent components analysis (ICA) for uncertain timing, or the Granger causality for effective connectivity, just to mention a few of the multiple possible analyses [7].

#### 2.1.2. Positron Emission Tomography (PET)

For imaging with a PET device, radiotracers are applied into the blood stream. At their destination, these tracers represent the brain's regional blood flow, oxygen, or glucose metabolism and reflect the activity in the according brain region. PET markers are very specific and imaging their effects is not limited in depth. However, the limited spatial resolution and its time-consuming and expensive procedure make PET less attractive than fMRI [8].

#### 2.1.3. Electroencephalography (EEG)

EEG typically measures the macrorhythms in the cortex, with impulses from subcortical structures. These rhythms are signals at frequencies below 100 Hz, reflecting primarily postsynaptic potentials [9] with high temporal resolution.

In addition, EEG measurements can also numerically describe the depth of sedation by assessing the bispectral index (BIS). BIS values in a range between 40 and 60 indicate general anesthesia [10].

#### 2.1.4. Evoked Potentials (EP)

In this review, somatosensory evoked potentials (SEPs) and auditory evoked potentials (AEPs) will be discussed. SEPs are activities of the somatosensory cortex after stimulation of peripheral nerves (e.g., median nerve), whereas AEPs are generated by sound, usually by clicks.

The amplitude of an EP measurement reflects the number of cortical cells activated and the magnitude of spatial summation of inhibitory postsynaptic potentials (IPSPs) and excitatory postsynaptic potentials (EPSPs) [11]. These present not only the intensity of the stimulus, but also the subjective experience [12]. Because of this, late SEP components might represent correlates of cognitive and evaluative stimulus processing [13].

### 2.2. Acupuncture Analgesia

#### 2.2.1. Perception of Pain

The perception of pain consists of a sensory and an affective component.

The sensory/spinothalamic pathway starts at peripheral nociceptors, which deliver the noxious stimulus via the spinal cord, brainstem, and thalamus to the somatosensory cortex to provide information about the location and intensity of the painful stimulus (primary (S1) and secondary somatosensory cortex (S2), sensory aspect: “there is a dull pain in my right hand”).

The affective component of pain is delivered via the spinobrachial pathway from the superficial dorsal horn to a network of amygdala, insula, anterior cingulate cortex (ACC, affective aspect: “it really hurts”), and medial regions of the frontal lobe (prefrontal cortex (PFC), cognitive aspect: “when will it stop?”) [14].

#### 2.2.2. Brain Regions Involved in Processing Pain

Several brain regions engage in processing the affective (amygdala, hippocampus), sensory (thalamus, S1 and S2), and cognitive (ACC, anterior insula) components of experiencing pain [15].

#### 2.2.3. Acupuncture Analgesia Mechanisms

Leung [16] claims that acupuncture analgesia might be induced by the release of endogenous opioids, by the modulation of the adrenergic system/the serotonin signaling system/the N-methyl-D-aspartic acid/AMPA/kainate signaling system, by the modulation of long-term depression and long-term potentiation of neural plasticity, or finally by the activation of the diffuse noxious inhibitory control system.

#### 2.2.4. Acupuncture Modulates Brain Regions

On top of the possible mechanisms of acupuncture analgesia mentioned above, more recent research proposes that acupuncture needling modulates certain areas of the pain matrix in the brain.

In 2007, Dhond et al. [5] presented a review about neuroimaging studies, which demonstrates that acupuncture modulates a wide network of brain areas including cortical, subcortical/limbic, and brainstem areas [17–22].

The review summarizes that after the first localization and characterization of the acupuncture stimulus in the somatosensory cortices (S1, S2), limbic brain regions like the hypothalamus, amygdala, cingulate, and hippocampus are recruited.

While hippocampus and amygdala are supposed to support learning and memory in pain pathways, the amygdala might encode the affective component of pain [23]. Additionally, Dhond et al. [5] propose that stress reduction by shifting autonomic nervous system (ANS) balance, affect, and cognition could be another possible benefit.

The paper also points to a further connection that links the hippocampus and amygdala with the brainstem and the hypothalamus. As the latter modulates neuroendocrine and homeostatic function, these interactions could possibly affect arousal and motivation.

Moreover, Dhond et al. [5] state that modulation of the anterior and posterior insula might also play a role in acupuncture effects, as these areas influence changes of attention and effect [22, 24], similar to the PFC, which, respectively, connects to the limbic system and modulates expectancy [25].

#### 2.2.5. Acupuncture Modulates Brain Networks

In the last years of fMRI research, many studies concerning acupuncture's effect on the CNS came across the influence of resting state networks in the brain. The most important of these networks is the default mode network (DMN), which consists of the PFC, posterior cingulate cortex (PCC), and precuneus as well as lateral, parietal, and temporal regions [26–28]. The DMN is active when the individual focuses on internal tasks.

In a review from 2012, Otti and Noll-Hussong [29] point out that the above-mentioned effects of acupuncture on the brain could possibly trace back to an enhanced functional connectivity between the DMN and several brain areas (including the hippocampus, periaqueductal gray (PAG), amygdala, and anterior cingulate).

This connection might explain why real acupuncture reintegrates balance of emotions, thinking, and the body.

## 3. Methods

### 3.1. Eligibility Criteria

For the literature research the following eligibility criteria were set.

#### 3.1.1. Types of Studies

This review includes all published randomized and nonrandomized controlled clinical studies as well as observational studies (cohort and case studies) with control groups, no blinding required. Inclusion of studies was restricted to English language.

Meta-analyses, reviews, and studies without control were not considered.

#### 3.1.2. Types of Participants

Only trials with 10 or more healthy participants of either gender, aged 18 or older, were included. Patients or people with any record of substance abuse or addiction were excluded.

#### 3.1.3. Types of Interventions

Only those studies were accepted in which at least one group received needle acupuncture at one or more acupuncture point, A-Shi or trigger points.

Needle acupuncture interventions refer to recommendations of the WHO 2002:

- manual acupuncture (MA): stimulation of points on the body through penetrating the skin with thin, solid, metallic needles that are manipulated by the hands,
- electroacupuncture (EA): passing a pulsed current through the body using acupuncture needles.

#### 3.1.4. Control Groups

Studies were included if the control group received any style of sham acupuncture stimulation, which did not intend to be a treatment.

However, groups comparing different kinds of needle acupuncture treatments and groups with no intervention or with any treatment unrelated to acupuncture point stimulation did not constitute an eligible control group.

Accepted sham acupuncture procedures were

- manual sham acupuncture with needle insertion: superficial penetration of the skin or needling at a NMP (nonmeridian point) or NAP (nonacupuncture point), even if performed with stimulation or manipulation,
- manual sham acupuncture without needle insertion: blunt needles or Streitberger needles,
- sham EA with disconnected electrodes,
- sham laser acupuncture with a switched off laser device,
- tactile stimulation of acupuncture points (comparable to blunt needling).

#### 3.1.5. Search Methods for Identification of Studies

The PubMed Database was initially searched from 1/1/2000 to 1/6/2014 with restriction to human studies in English language.

During the course of the study, data collection for fMRI studies was restricted to the time from 1/1/2010 to 1/6/2014 due to a recently published review.

PubMed sources were searched with the following medical subject heading terms and search strategies:

- ((fMRI) OR (MRI, Functional) OR (Functional MRI) OR (Functional MRIs) OR (MRIs, Functional) OR (Magnetic Resonance Imaging, Functional)) AND acupuncture.
- ((Positron Emission Tomography) OR (PET Scan) OR (PET Scans) OR (Scan, PET) OR (Scans, PET) OR (Tomography, Positron-Emission) OR (Tomography, Positron Emission)) AND acupuncture.
- ((EEG) OR (Electroencephalogram) OR (Electroencephalograms)) AND acupuncture.
- ((Evoked Potential) OR (Potential, Evoked) OR (Potentials, Evoked) OR (Potentials, Event-Related) OR (Event-Related Potential) OR (Potential, Event-Related) OR (Potentials, Event Related) OR (Event-Related Potentials) OR (Event Related Potentials)) AND acupuncture.

*Searching Other Resources.* References of selected publications and bibliographies of reviews (found during the first screening of publications) were inspected for more potentially useful articles.

### 3.2. Study Selection

Title and abstract of all results in the literature search list were examined and full texts were retrieved if possible.

During the first screening duplicates, reviews and studies with unrelated topics were removed as well as any studies where full text was not available.

In the second step full texts of potential studies were evaluated according to predefined inclusion and exclusion criteria.

### 3.3. Data Collection

Data of all included studies were extracted with regard to the STRICTA (standards for reporting interventions in clinical trials) guidelines. The considered study characteristics and variables were then transferred to Excel. 

*Publishing Data:*

- author,
- year,
- title,
- journal.

*Methodology:*

- number of participants,
- participants' handedness,
- number of intervention groups,
- number of treatment sessions.

*Needling Details:*

- acupuncture rationale: manual/electro,
- acupuncture points (uni-/bilateral),
- needling depth,
- style of manipulation,
- response elicited (De-Qi),
- needle retention time.

*Control Intervention:*

- types of control intervention,
- size of intervention groups.

*Technology:*

- neuroimaging method,
- technical device,
- data processing software.

*Objective/Outcome:*

- objective,
- findings,
- group differences,
- increase/decrease, activation/deactivation.

### 3.4. Subgroup Analyses and Assessment of Heterogeneity

Within neuroimaging groups with sufficient data, subgroup analysis was applied to evaluate the differences of MA versus EA in all kinds of neuroimaging, meaning fMRI, EEG + EP, and PET.

In addition, results of studies needling the same point(s) were compared within their respective imaging intervention group.

## 4. Results

### 4.1. Study Selection

Literature search was conducted from 1/12/2013 to 1/3/2014. From 1/3/2014 updates were signed up and followed by PubMed newsletter.

For the first screening, the search period ranged from 1/1/2000 to the end of May 2014. Later during the course of the study, data collection for fMRI trials was restricted to a narrower time frame ranging from 1/1/2010 to 1/6/2014 due to a recently published review.

**Figure 2:**
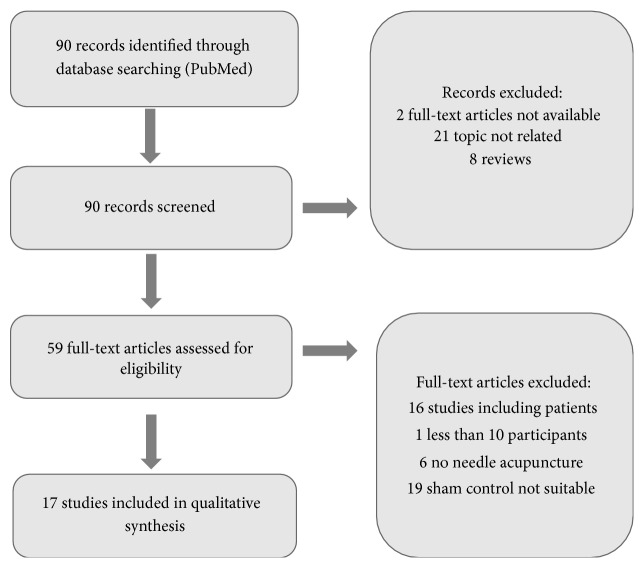
Flowchart of screening fMRI studies.

**Figure 3:**
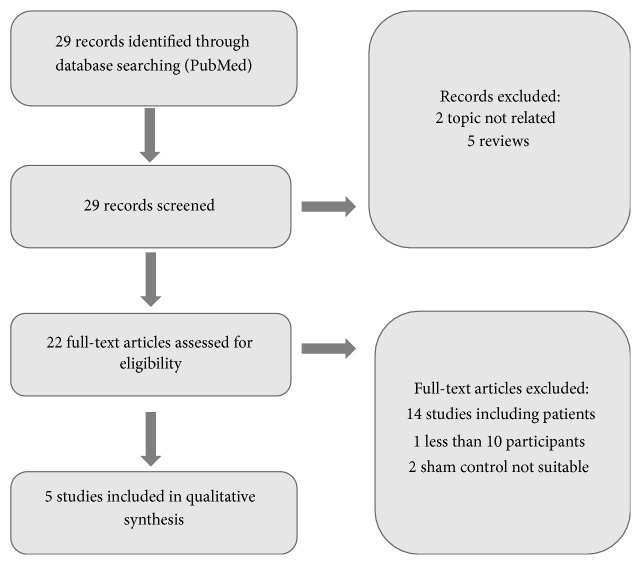
Flowchart of screening PET-studies.

**Figure 4:**
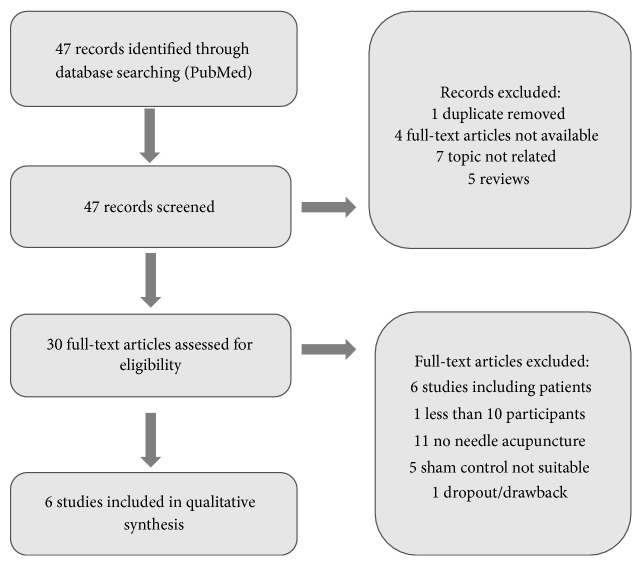
Flowchart of screening electroencephalogram studies.

**Figure 5:**
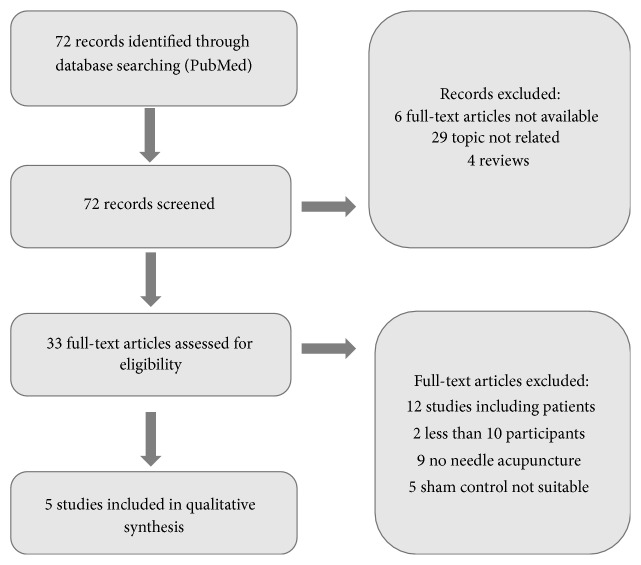
Flowchart of screening EP-studies.

The study selection process is illustrated in Figures 2, 3, 4, and 5.

### 4.2. Total Studies Considered in This Review Paper

In total, the first PubMed Database search resulted in 238 studies about acupuncture and neuroimaging—including all categories (fMRI, EEG, EP, and PET).

Out of the 238 studies, 90 were found in the field of acupuncture and fMRI, 47 in EEG, 72 in EP, and 29 in PET.

After removing 94 duplicates, reviews, studies with unrelated topics, and trials where no full text was available, a total of 144 papers were included for further evaluation—including 59 fMRI, 30 EEG, 33 EP, and 22 PET trials.

During the second screening, these 144 potential studies were evaluated according to predefined eligibility criteria. After exclusion of another 111 trials, this step resulted in the final inclusion of 33 studies on acupuncture and neuroimaging, comprising 17 fMRI, 6 EEG, 5 EP, and 5 PET studies.

### 4.3. Studies Excluded from the Review

In the process of selection, a total number of 205 studies were excluded.

During the first course of eligibility screening 94 duplicates, reviews, studies with unrelated topics, and trials where no full text was available were excluded.

Further on, the second evaluation excluded another 111 studies due to the following reasons: study included patients, number of participants < 10, no group received needle acupuncture, or no suitable control group included.

### 4.4. Studies Included in the Review

PubMed database screening revealed 238 studies about neuroimaging acupuncture effects, including 90 fMRI, 47 EEG, 72 EP, and 29 PET trials.

After evaluation according to the exclusion criteria above, a total number of 33 studies on acupuncture and neuroimaging were included in this review, comprising 17 fMRI, 6 EEG, 5 EP, and 5 PET studies.

### 4.5. Participants

33 studies with a total number of 687 participants were selected, comprising 399 participants in fMRI, 72 in PET, 99 in EEG, and 117 in EP trials.

All included papers stated their inclusion and exclusion criteria clearly enough to enable this review to only consider healthy adult volunteers. In addition to general data extraction, participants' handedness was considered in all fMRI studies.

### 4.6. Sample Size

All studies had a sample size of at least 10 participants per study, with a range from 6 to 25 participants per intervention group.

### 4.7. Interventions

#### 4.7.1. Main Interventions: MA and EA

Distribution of the two main interventions MA and EA varied significantly between the studies of different neuroimaging methods.

Out of 33 studies, 25 applied MA versus sham, only 7 applied EA versus sham. One study compared EA versus MA.

All 5 EP studies applied EA and compared EA versus sham acupuncture [30–34]. All 5 PET studies applied MA versus sham acupuncture [17, 24, 35–37]. Five out of 6 EEG studies applied MA versus sham acupuncture [38–42]; one EEG study used EA versus sham acupuncture [43]. Fifteen out of 17 fMRI trials applied MA versus sham acupuncture [44–58]; one fMRI study used EA versus sham acupuncture [59], and one fMRI trial compared EA versus MA [60].

#### 4.7.2. Needling Depth

Needling depth varied significantly throughout all studies, ranging from 0.3 mm (EEG, [43]) up to 3 cm (fMRI, [44, 50, 53, 55]). Mean needling depth of the available 25 studies was 14.03 mm. Eight studies did not report details about needling depth (fMRI, [45, 47–49]; EP, [30, 32, 33, 38]).

#### 4.7.3. Acupuncture Points

Out of the 33 trials, 28 only chose one single acupuncture point, 5 used a combination of two points, and 3 studies applied a combination of 3 or more points. Point selection varied significantly and included points on both arms, both legs, and the head.

Altogether 50 points were reported. Their application was distributed as follows: ST36, *n* = 13; LI4, *n* = 10; PC6, *n* = 4; LV3, *n* = 4; GB37, *n* = 3; SP6, *n* = 2; SP9, *n* = 2; TH5, *n* = 2; and Yintang, *n* = 2. Points used in one study only were LI3, LI11, BL60, BL62, LU5, PC5, HT7, and Ear Shenmen.

#### 4.7.4. Needle Retention Time

30 studies reported the needle retention time of their main interventions. The average duration was approximately 12 min.

#### 4.7.5. Control Interventions

As listed below, most of the studies (29 out of 33) only used one single control intervention.

Non-acupuncture-points (NAP) were used 20 times: fMRI: 11 studies with MA versus NAP [44, 45, 47–53, 57, 58]; PET: 3 studies with MA versus NAP [17, 24, 37]; EEG: 1 study with EA versus NAP [43]; EEG: 3 studies with MA versus NAP [38–40]; and EP: 2 studies with EA versus NAP [33, 34].

Streitberger needles were used 3 times: fMRI: EA versus Streitberger sham EA [59]; PET: MA versus Streitberger sham [35]; and EEG: MA versus Streitberger sham [42].

Von Frey filaments were used twice: fMRI: MA versus von Frey [55] and fMRI: MA versus von Frey versus tactile stimulation [54].

Overt sham with blunt needling was used once: fMRI: MA versus blunt needle [56].

Painful tactile stimulation with cotton tip at an acupuncture point was used once: fMRI: MA versus tactile stimulation [46].

Sham EA with no needle but electro tape was used twice: EP: EA versus sham EA [31, 32].

Five out of 33 studies used the following combinations of control interventions: EEG: NAP acupressure versus manual versus laser [41]; EP: NAP versus electro without deqi versus with deqi versus painful overstimulation [30]; PET: Streitberger versus MA versus overt blunt needling [36]; and fMRI: von Frey filament versus MA versus EA versus transcutaneous electrical acupoint stimulation (TEAS) [60].

### 4.8. Study Objectives and Outcomes

Tables 1–4 present an overview of the outcomes of the studies included in this review.

### 4.9. Result Tables

A comparison of all included studies with regard to technical devices used, control intervention, number of participants, and so forth can be found in Tables 5, 6, 7, and 8.

### 4.10. Subgroup Comparisons

#### 4.10.1. Comparison of Main Interventions

*(1) MA versus EA (fMRI: Zyloney et al., 2010 [60])*

- Of the 33 studies, only one fMRI study compared MA versus EA. Zyloney et al. [60] investigated the spatial and temporal effects of manual, EA, and TEAS at ST36 at the left leg.
- By using a modified generalized linear model analysis to compare block-designed and resting-state fMRI scans they detected positive activation in the sensorimotor areas and negative activation in the default mode areas in both of the two 1 min simulation periods for tactile stimulation with a von Frey filament and in the first 1 min stimulation of MA, EA, and TEAS. However, in the second 1 min stimulation period, no positive activation result was observed and EA showed a more extensive deactivation compared to MA and TEAS.
- All modalities increased the instinct brain network in rest. A more secure and spatially extended connectivity of the DMN was observed following MA and EA, whereas TEAS specifically increased the functional connectivity in the sensorimotor network.

#### 4.10.2. Comparison of Verum Acupuncture versus Sham

*(1) Comparison of EA versus Control Group*

*(a) EA versus Streitberger Sham EA (fMRI: Liu et al., 2011 [59])*

- Out of 7 EA studies, only one study used Streitberger needles for sham EA. Liu et al. [59] analyzed the functional connectivity of the PAG during real EA and sham EA at LI3 and LI4 on the right hand in volunteers with high and low expectancy.
- They found greater connectivity between the PAG, left PCC, and precuneus in the comparison of verum EA versus Streitberger sham EA, whereas there was greater connectivity in the PAG and right anterior insula for sham EA. No significant differences were observed between high and low expectancy groups.

*(b) EA versus Sham EA with Tapes/Patches (EP: Kvorning et al., 2003 [31]; Meissner et al., 2004 [32])*

- Two studies observed the influence of EA versus sham EA on EPs. Kvorning et al. [31] investigated the effects on AEPs and Meissner et al. [32] investigated changes of SEPs.
- Kvorning et al. [31] explored the effects on AEPs of bilateral verum EA versus sham EA at LI4, PC6, ST36, SP9, LR3, and SP6 in anesthetized participants. However, they found no significant difference of (mid-latency or any other) AEPs between the two groups, which could have correlated with the depth of anesthesia.
- Meissner et al. [32] evaluated SEP changes after bilateral verum EA versus sham EA at ST36 and LR3 in anesthetized volunteers. They detected a decrease in the magnitudes of late SEP amplitudes (P260) after verum but not sham EA.

*(c) EA versus NAP (EEG: Kim et al., 2009 [43]; EP: Wei et al., 2000 [33]; and Zeng et al., 2006 [34])*

- Three trials studied the differences of verum EA versus EA at a nearby NAP. Kim et al. [43] investigated the effects on the EEG, whereas Wei et al. [33] inspected changes of SEPs. Zeng et al. [34] combined temporal examination of EEG activities and SEP changes.
- As studies comparing acupuncture at a certain acupuncture point versus NAP mostly aim at neuroimaging point specific effects on the CNS, this subgroup analysis will only be discussed below, where trials using one single acupuncture point will be grouped according to the point they investigated. For Kim et al. [43] please refer to Table 3, and for Wei et al. [33] and Zeng et al. [34] please refer to Section 4.10.3 *Point specificity comparison*.

*(2) Comparison of MA versus Control Group*

*(a) MA versus Overt Painful Tactile Stimulation (fMRI: Cho et al., 2010 [46])*

- The fMRI study by Cho et al. [46] compared manual versus overt painful tactile stimulation with a cotton tip at LI11 on the left arm versus ST36 on the left leg.
- In comparison to painful tactile stimulation, MA at LI11 led to activation of both sides of the parahippocampal gyrus, cerebellum, left side of thalamus, and right side of posterior cingulate regions.
- Acupuncture but not tactile stimulation at ST36 produced activation at the S2, limbic system (cingulate gyrus, posterior cingulate), V1, pons, medulla regions at the left BA 6, BA 8, and ACC.
- In comparison with the left LI11 acupuncture stimulation, left BA 6, BA 8, and ACC were more activated by the left ST36 acupuncture stimulation.
- Acupuncture activated more regions than painful tactile stimulation, especially areas of the limbic system, such as the parahippocampal gyrus and ACC.

*(b) MA versus Overt Blunt Needling (fMRI: Yeo et al., 2010 [56])*

- Yeo et al. [56] focused on investigating the effect of previous acupuncture stimulations on brain activations of later acupuncture stimulations.
- They found that after the first verum acupuncture stimulation block at the left BL62, the left hemisphere showed activation in the hypothalamus, thalamus, claustrum, cerebellum, inferior frontal gyrus, and the superior temporal gyrus, while the right hemisphere presented activation in the middle frontal gyrus. In both hemispheres, a significant focus of activation was found in the inferior parietal lobule.
- During the second block, only the cerebellum in the left hemisphere and the inferior parietal lobule in the right hemisphere were significantly activated, showing decreased activations during the second verum acupuncture stimulation.

*(c) MA versus Von Frey Filaments (fMRI: Murase et al., 2013 [54]; Napadow et al., 2013 [55])*

- Two studies observed the effects of MA versus tactile stimulation with von Frey filaments on the fMRI. Both studies examined spatial and temporal effects of acupuncture, but Napadow et al. [55] focused mainly on ANS responses.
- Murase et al. [54] investigated the fMRI effects of MA versus von Frey filament stimulation at LI4 on the right hand versus touch stimulation at the right palm with a deconvolution analysis with Tent functions.
- MA showed activation on both sides in the S2 and the insula, on both sides in the S1, the M1, ACC, SMA, thalamus, and PFC.
- Sham acupuncture with von Frey filament showed activation in the contralateral S1 and SMA and on both sides in the S2 and insula. Tactile stimulation showed activated areas in the contralateral S1, M1, and SMA and on both sides in the S2 and insula.
- Real acupuncture induced more widespread, more delayed, and long-sustained increases and decreases of BOLD signal in the somatosensory region and in areas related to pain perception.
- Napadow et al. [55] combined fMRI with several interventions to measure the ANS response to MA on the left leg at ST36 versus SP9 versus tactile stimulation with von Frey filaments at a NAP near ST36.
- GLM measurements showed that acupuncture events with strong skin conductance response produced greater anterior insula activation and acupuncture at SP9, which produced greater skin conductance response, also produced stronger sharp pain sensation and greater anterior insula activation.
- Acupuncture-induced HR deceleration was associated with greater DMN deactivation. This association was strongest for ST36, which produced more robust HR deceleration.
- DMN deactivation was significantly more pronounced across acupuncture stimuli producing HR deceleration versus those events characterized by acceleration.

*(d) MA versus Streitberger Needles (PET: Dougherty et al., 2008 [35]; EEG: Streitberger et al., 2008 [42])*

(i) Two trials compared MA and Streitberger needles sham acupuncture.
(ii) Dougherty et al. [35] used PET and Streitberger et al. [42] applied quantitative EEG (qEEG) to view acupuncture's effects on the brain.
(iii) Dougherty et al. [35] studied the binding of PET opioid agonists and according fMRI changes after MA versus Streitberger needle acupuncture at LI4 on the right hand.
(iv) In comparison to Streitberger acupuncture, they observed significant changes during verum acupuncture in the medial and lateral pain networks, such as opioid-binding decreases (associated with greater endogenous opioid release) in the right OFC, left medial PFC, right insula, and right thalamus, as well as binding increases in the bilateral insula, right medial PFC/ACC, left OFC, and right brainstem.
(v) An overlap of results between fMRI signals and [11C] diprenorphine blood pressure changes was only exhibited in the right medial OFC.
(vi) Streitberger et al. [42] examined the quantitative effects of bilateral MA at LI4 versus Streitberger needle acupuncture at a nearby NAP on the qEEG.
(vii) In linear relation to HRV changes, verum acupuncture influenced the power EEG with increase in the alpha1-frequency of the occipital region with a shift of the alpha1/theta ratio to the benefit of alpha1 over all electrodes.
(viii) A negative linear correlation was found between the theta-band of the qEEG and the HRV parameters, and a negative linear correlation was also found between low frequency and alpha1 as well as between high frequency and alpha1.

*(e) MA versus NAP*

- A total of 17 trials studied the differences of verum MA versus MA at a nearby NAP.
- Three studies investigated changes with PET (Biella et al., 2001 [23]; Hsieh et al., 2001 [17]; Schlünzen et al., 2007 [37]), and another three studies applied EEG (Cabrini et al., 2006 [38]; Hsu et al., 2011 [39]; and Kim et al., 2008 [40]) and the majority of eleven studies compared the fMRI effects of manual acupuncture versus acupuncture at NAP (Bai et al., 2010 [44]; Cheng et al., 2013 [45]; Dong et al., 2012 [47]; Feng et al., 2011 [48]; Li et al., 2010 [49]; Liu et al., 2010 [50]; Jiang et al. 2013 [58]; Liu et al., 2012 [51]; Liu et al. 2012 [52]; Liu et al., 2013 [53]; and You et al., 2013 [57]).
- As studies comparing acupuncture at a certain acupuncture point versus NAP mostly aim at neuroimaging point specific effects on the CNS, this subgroup analysis will only be discussed below, where trials using one single acupuncture point will be grouped according to the point they investigated.
- For Biella et al. (PET) [24]; Cabrini et al. [38] and Hsu et al. [39] (EEG); Kim et al. [40]; and Liu et al. [52] (fMRI) please refer to the Tables 1, 2, and 3.
- For Hsieh et al. [17], Schlünzen et al. [37] (PET) and Bai et al. [44], Cheng et al. [45], Dong et al. [47], Feng et al. [48], Li et al. [49], Liu et al. [50], Jiang et al. [58], Liu et al. [51], Liu et al. [53], and You et al. [57] (fMRI) please refer to Section 4.10.3 *Point specificity comparison*.

*(3) Verum versus Combined Control Interventions*

*(a) EA with deqi versus without deqi versus Painful Stimulation versus NAP (EP: Abad-Alegría and Pomarón, 2004 [30])*

- Abad-Alegría and Pomarón [30] investigated SEP changes due to EA at LI4 during different time points of needling at LI4, including puncturing the skin without deqi and needling with deqi as well as painful overstimulation, versus EA at a NAP.
- Their measurements showed a direct relation between F-waves and SEPs with increasing electrostimulus, with main inflexion during deqi, whereas, with ongoing stimulation, greater variations took place, especially in case of SEP latency.
- In contrast, EA at a NAP did not produce any of the aforementioned effects.

*(b) MA versus Several Control Interventions*

- MA versus EA versus TEAS versus von Frey filament (Zyloney et al. [60]) refer to Section 4.10.1(1)* (MA versus EA*).
- Litscher compared the effects of MA versus laser acupuncture versus acupressure at Yintang versus acupressure at a nearby NAP on the BIS.
- The study reports a decrease of BIS and spectral edge frequency values for acupressure and laser acupuncture at Yintang and for acupressure at the NAP but not for manual acupuncture.
- Lai et al. [36] evaluated CBF changes with 18-fluoride-deoxyglucose PET during MA versus overt blunt needling versus sham blunt needling (similar to a Streitberger needle) of TH5 on the right arm.
- For MA in comparison to overt blunt needling, more brain areas (BA7, 13, 18, 19, 21, 22, 27, 38, 40, 42, and 45) were activated, whereas, in comparison with Streitberger-like sham acupuncture, slightly less MA activation was found in the areas of BA13 and 42.
- During Streitberger-like sham acupuncture the areas BA4, 6, 7, 19, 22, and 41 showed activation.

#### 4.10.3. Point Specificity Comparison

Out of the 33 trials, 28 chose needling at only one single acupuncture point.

If within the same neuroimaging group there was more than one study about a single acupuncture point in comparison to another point or to a NAP, these studies were compared. In total, this resulted in the comparison of 15 studies, comprising 3 studies on GB37, 5 studies on LI4, and 7 studies on ST36.

*(1) GB37.* In total, three fMRI studies investigated acupuncture at GB37 versus NAP. Li et al. [49] and Liu et al. [53] compared MA at GB37 versus NAP. Dong et al. [47] additionally compared MA at GB37 with MA at BL60 versus acupuncture at NAP.

- Dong et al. [47] aimed at studying the temporal fMRI effects of MA at the vision-related acupuncture points GB37 versus BL60 versus a nearby NAP.
- Although the ICA of all kinds of acupuncture showed activity at the V1 in the occipital lobe, temporal activities in this region differed for acupuncture at GB37 versus NAP, as well as for BL60 versus NAP.
- Li et al. [49] focused on distinguishing the fMRI effects of MA at GB37 versus NAP with multi-voxel pattern analysis (MVPA).
- They found different effects for verum acupuncture versus NAP in the subregions of occipital cortex (left cuneus of occipital gyrus and regions of lingual gyrus, middle occipital gyrus and fusiform gyrus), the limbic-cerebellar system (including insula, rACC and pACC, pons, amygdala, culmem in anterior lobe and declive of vermis in posterior lobe of cerebellum), and the somatosensory cortex.
- For GLM, the neural response patterns of acupuncture stimulation at acupoint and NAP had multiple overlapping regions and did not significantly differ from each other.
- Liu et al. [53] examined the different spatial and temporal effects of MA at GB37 versus NAP.
- GLM analysis showed a more extensive spatial distribution signal decrease in the limbic-cerebellar regions (such as the occipital cortex, pons, PH/Hipp, putamen and cerebellum), but with a smaller signal increase (such as in the STG, S2 and thalamus).
- Special temporal investigation showed that the neural response evoked by acupuncture did not turn on and off rapidly but lasted longer, violating the basic assumption of standard GLM analysis.
- fMRI signals of the limbic-paralimbic-neocortical system increased, so that changes in the occipital cortex showed different temporal patterns between GB37 and NAP.

*(2) LI4*

*(a) Two PET Studies Compared Acupuncture at LI4 versus NAP*

- Hsieh et al. [17] compared MA at LI4 with acupuncture at a nearby NAP and Schlünzen et al. [37] compared MA with acupuncture at a NAP in anesthetized participants.
- Hsieh et al. [17] studied point specific changes of CBF during MA at LI4 on the right hand versus a nearby NAP.
- In comparison to acupuncture at a NAP, only MA at LI4 elicited activation of the rCBF in the areas of the hypothalamus with extension to midbrain, the insula, the ACC, and the cerebellum.
- In addition, a further comparison of needling with deqi contrasted with minimal manipulation acupuncture and showed activation in the hypothalamus and the cerebellum. The activation by deqi in the hypothalamus extended to the midbrain/brainstem when contrasted with the brain at rest. Minimal stimulation activated neither the hypothalamus nor the insula when compared with rest situation.
- Schlünzen et al. [37] also observed point specific changes of CBF during MA at LI4 on the right hand versus a nearby NAP. Different from Hsieh et al. [17], the study participants were anesthetized prior to acupuncture treatment.
- Their results showed a decrease in CBF in the right medial frontal gyrus and in the left putamen for verum acupuncture. Acupuncture at a nearby NAP only caused a decrease of CBF in the right medial frontal gyrus.

*(b) Three EP Studies Explored EA Effects at LI4 versus NAP*

- Abad-Alegría and Pomarón [30] applied EA without deqi and with deqi at LI4 and compared it to a nearby NAP. Wei et al. [33] only inspected changes of SEPs whereas Zeng et al. [34] combined temporal examination of EEG activities and SEP changes after EA at LI4 versus NAP.
- Abad-Alegría and Pomarón [30] 2004 investigated SEP changes due to EA at LI4 during different time points of needling at LI4, including puncturing the skin without deqi and needling with deqi as well as painful overstimulation, versus EA at a NAP.
- Their measurements showed a direct relation between F-waves and SEPs with increasing electrostimulus, with main inflexion during deqi, whereas, with ongoing stimulation, greater variations took place, especially in case of SEP latency.
- In contrast, EA at a NAP did not produce any of the aforementioned effects.
- Wei et al. [33] examined SEPs elicited by verum EA at LI4 on the right arm versus EA at a nearby NAP in comparison to median nerve stimulation.
- Their results presented longer N1 and N2 latencies by MA at LI4 as well as acupuncture at a nearby NAP than by median nerve stimulation but showed no significant SEP differences between MA at LI4 versus NAP.
- Zeng et al. [34] evaluated EEG activities after EA at LI4 versus NAP and the acupuncture effects on painful SEPs of median nerve stimulation.
- EA at LI4 but not at a nearby NAP produced later-latency SEPs (P150) in bilateral ACC and attenuated pain specific amplitudes of P170 and N280 after median nerve stimulation.

*(3) ST36.* Seven fMRI studies compared MA effects at ST36 with MA at a nearby NAP (Bai et al., 2010 [44]; Cheng et al., 2013 [45]; Feng et al., 2011 [48]; Liu et al., 2010 [50]; Jiang et al., 2013 [58]; Liu et al., 2012 [51]; and You et al., 2013 [57]).

- Bai et al. [44] investigated the temporal effects of MA at ST36 versus NAP with a nonrepeated event-related (NRER) fMRI paradigm and change-point analysis.
- They found that the amygdala and pACC exhibited increased activities during needling but decreased to reach a peak below the baseline. The PAG and hypothalamus presented intermittent activations across the whole session.
- Apart from the time-dependent responses, relatively persistent activities were also identified in the anterior insula and PFCs.
- In comparison, verum and sham shared a similar activation pattern in somatosensory areas (S1 and S2) during needling. However, during the postacupuncture resting period acupuncture at ST36 was followed by sustained activation of the S2, whereas acupuncture at NAP showed inhibition of the S1.
- Cheng et al. [45] applied graph theoretical analysis with pairwise correlations of cortical and subcortical regions to evaluate NRER fMRI effects of manual acupuncture.
- Their correlations presented frequency-specific modularity functional brain networks during poststimulus resting state following acupuncture at ST36 and NAP.
- Graph metrics in brain activity are different in verum and sham groups and also show that the brain network following MA has higher global and local efficiency in parallel information transfer in the brain network compared with acupuncture at a NAP.
- Feng et al. [48] evaluated interaction and changes of large scale networks after MA at ST36 versus NAP.
- Within a network of 90 predefined regions in the poststimulus resting brain, limbic/paralimbic regions (such as the amygdala, hippocampus, and ACC) emerged as network hubs after verum but not sham acupuncture.
- Compared with needling at a NAP, MA at ST36 presented increased correlations, related with the limbic/paralimbic and subcortical regions (such as the insula, amygdala, and ACC) and thalamus. Decreased correlations for verum acupuncture were related with the sensory and frontal cortex.
- Liu et al. [50] studied the spatial and temporal effects of MA at ST36 versus NAP in a nonevent-related paradigm with GLM and ICA.
- Their results showed manipulation-related effects and sustained acupuncture effects in the cortical-subcortical areas, including the ACC, VLPFC, and SMA, and decreases in the S1 and S2.
- These reactions lasted until the resting period after needling, where then activations were induced in many regions including the insula, caudate, putamen, and thalamus.
- Liu et al. [58] examined the immediate and delayed effects of acupuncture at ST36 versus NAP with GLM and graph theory analysis.
- The immediate effect of verum as well as sham acupuncture consisted of signal changes in the limbic/paralimbic areas, neocortical regions, brainstem, and cerebellum.
- For a delayed effect, several regions showed strong functional connectivity. During the overall process of acupuncture, the insula played a critical role.
- Acupuncture at NAP produced positive activations with a small extent of spatial distribution and less intensive signal change as compared to ST36, mainly in the insula, S2, and cerebellum.
- Liu et al. [51] focused on searching the spatial effects of MA at ST36 versus NAP with applying small-world brain networks.
- The results presented increased local efficiency after acupuncture stimulation. No significant differences were found for sham acupuncture at a NAP. Significant effects of real acupuncture but not sham were detected on nodal degree of the left hippocampus.
- Point-related effects were observed in the ACC, frontal, and occipital regions while stimulation-related effects were found in various brain regions of frontal, parietal, and occipital cortex regions. Several limbic and subcortical brain regions exhibited point- and stimulation-related alterations in their regional homogeneity.
- You et al. [57] used pairwise functional connectivity analysis in 8 regions of the DMN with 5 conventional frequency bands (delta, theta, alpha, beta, and gamma) to investigate band-specific alterations of DMN hub configurations after MA at ST36 on the right leg versus NAP.
- They found that after sham acupuncture at NAP, the PCC remained to serve consistently as DMN hub across all 5 frequency bands.
- However, the PCC was regulated and only acted as a DMN hub within delta and gamma bands after verum acupuncture at ST36.
- 28 of the 33 trials were further included into the subgroup analysis and assigned to one of the three main groups: A = comparison of main interventions, B = verum acupuncture versus sham acupuncture, and C = point specificity (see Table 9).

In conclusion, it was not reasonable to further compare the results within their subgroups due to the heterogeneity of studies, with different research questions and diverse methodology protocols. In Table 9, the outcomes shall therefore only be listed as short descriptive results.

## 5. Discussion

### 5.1. Summary of Evidence

In the following, the results of all 33 trials included in this review will be presented in groups according to their neuroimaging intervention and outcome.

Out of 17 fMRI studies, one found greater connectivity for verum EA, 5 found changes of network efficiency or network correlations, 9 found activation of different areas, and 6 found different temporal changes with verum MA.

Out of 5 PET studies, 3 found more activation of CBF, one found a greater release of endogenous opioids, and one found a decrease of CBF with verum MA.

Out of 6 EEG studies, 2 found no changes of BIS with verum MA and 4 found EEG signal increases in different bands with verum acupuncture (3 MA and 1 EA).

Out of 5 EP studies with verum EA, one found no influence on AEPs, one found no difference in acupuncture or sham SEPs, and 3 found an influence on late SEPs.

### 5.2. Limitations

Although this review paper provides a detailed structured overview of the current literature with respect to neuroimaging and acupuncture, the data evaluation encountered typical, all too well known difficulties in acupuncture mechanism research due to heterogeneous methodology protocols.

While conducting studies about acupuncture's neurological mechanisms, every research question, every neuroimaging device, and every verum and control acupuncture intervention holds its own risk of bias. Therefore, a few of the most important limitations of the studies included in this review shall be addressed in the following.

#### 5.2.1. Methodology Protocol

*(1) Different Interventions: EA versus MA.* So far, several neuroimaging studies compared the effects of MA and EA on the brain and many of their findings were different. These differences should be addressed and kept in mind while choosing and discussing various interventions.

Several clinical studies made the assertion that EA shows stronger analgesic effects than MA [61, 62].

In addition, Hsieh [63] observed different ANS outflows for 2 Hz-EA versus MA, with larger latency and smaller amplitude of sympathetic skin response for EA than MA.

Several years later, Kong et al. [64] showed that EA mainly elicited fMRI signal increases in several brain areas, whereas MA produced strong decreases of fMRI signals.

One year later, Li et al. [65] found that different brain areas were activated by MA and EA and by EA of different frequencies.

Napadow et al. [21] furthermore extended these findings, by observing that EA elicited fMRI signal increases in more brain areas than MA.

Finally, a review by Huang et al. [66] stated that EA produces more activation and less deactivation compared to MA.

*(2) Different Acupuncture Points.* For clinical treatment, different doctors choose different individualized points for each patient. These treatment programs also vary based on different clinical experiences, styles, and schools. Above this, studies showed that even if the same points were chosen in theory, point locations still varied between doctors [67].

For research, so far no guidelines exist if, next to classical acupuncture points, A-Shi points or trigger points could also be considered real acupuncture points with similar mechanism of action. A far bigger problem still remains which is the fact that many studies even found similar effects for sham and real acupuncture points [68].

Referring to this, sham acupuncture points also should be chosen very carefully. Until now, no unified standards for locating NAPs exist. As there are many extra acupuncture points besides the meridians, sham points should best be chosen at nonacupoint, nonmeridian, and nonsegmental places [69].

*(3) Acupuncture Needling Depth.* In this review a broad range of needling depth was observed with big differences even for the same acupoints. With such striking differences, influences on the results are very likely and therefore detailed documentation is very important.

As White et al. [70] stated, needling depth differs on condition, age, and body weight. Also, different depth applies for safety issues of various acupuncture points [71].

Regarding the above statements, no generalization of advisable needling depth is possible. However, a study by Ceccherelli et al. [72] found that, for pain conditions, deep needling was superior to shallow needling.

Park et al. [73] reported different sensations according to the needling depth. This trial found that superficial penetration produced prominent pricking and sharp sensations, whereas deep penetration produced a high degree of deep, dull, heavy, spreading, and electric shock sensations.

Moreover, Zhang et al. [74] reported that deep needling produced more fMRI activation signals in almost all brain areas. In contrast, Wu et al. [18] found more activation from superficial needling in the somatosensory area, motor area, and language areas and more deactivation in the limbic system for deep needling.

*(4) Manipulation or Electrical Stimulation.* While the effects for electrical stimulation of different frequencies are well known since Han's publication in 2003 [75], less congruent data exist about the differences in strength, duration, and repetition of manual stimulation [75].

In clinical practice, various ways of stimulation can be applied, ranging from single stimulation and immediate pullout (single acupuncture stimulation) to keeping a needle inserted for some time (retaining needle) and continuous rotation of a needle with vertical movements during insertion (sparrow pecking method) [76].

Sakai et al. [77] found that rotation of a needle with vertical movements produced a stronger stimulation than holding or single stimulation.

Furthermore, Marcus [78] found that not only the style, but also the time of stimulation could make a difference. The study recorded that longer duration of manual stimulation contributed much more to the strength of acupuncture than the depth or gauge of the needle. Similarly, Li et al. [79] showed that longer duration of manipulation induced more activation of brain areas.

In addition, Zaslawski et al. [80] also confirmed that needling manipulation produced increased pain thresholds, even if a NAP was needled.

A newer fMRI review by Huang et al. [66] confirmed these statements as it found more activation in several brain areas for acupuncture with stimulation compared to no stimulation.

However, all of the above effects should always be investigated carefully, as repetition of stimulation causes the so-called “carryover effect,” which might lead to different results after several treatments [56, 81, 82].

*(5) Treatment Duration/Needle Retention Time.* In addition to considering the time of stimulation as discussed above, it is also important to think about the complete duration of needling, with or without stimulation.

For clinical treatment standard needle retention time averages 20 to 30 minutes [83]. He [84], Cheng et al. [85], and Peng and Fei [86] stated that practitioners should first wait for qi arrival after placing the needle. In their opinion, afterwards no further limitations for treatment time apply, once deqi was achieved.

In contrast, is more difficult to decide on the best needle retention time for research protocols. This question should be approached from different perspectives. From a theoretical point of view, researchers could consider the optimum time to release neurotransmitters after needle application [87], while practically needle retention time has to be adapted to the limitations and conditions of the respective neuroimaging intervention (e.g., block designs for fMRI, measurement time for EEG and BIS, etc.).

*(6) Deqi.* In traditional Chinese medicine (TCM) the deqi sensation is considered to be related to clinical efficacy [88].

From an anatomical viewpoint, different types of nerve fibers conduct different types of sensation. Soreness, dull pain, and heat sensations are transmitted by slow conductive, A-delta and C fibers, whereas numbness and tingling are transmitted by A-beta/gamma fibers. Pressure is transmitted through multiple different types of nerve fibers [83].

In an early study, Wang et al. [89] found numbness to be mostly associated with A-beta/gamma fibers, distension and heaviness with A-delta fibers, and soreness with C fibers [89].

Hendry et al. [90] furthermore stated that as A-beta fibers terminate in lamina III to VI of the dorsal horn and A-delta fibers terminate on large cells in lamina II, different acupuncture modalities might trigger different brain networks based on the types of afferent input.

According to this, deqi might vary in different treatment settings, depending on styles of acupuncture and stimulation. Park et al. [91] found that deqi from MA was slightly different from that of EA, but deqi from sham acupuncture was significantly lower than that of MA or EA. In contrast, Kong et al. [64] reported the difference that deqi of MA mainly induced soreness, fullness, and distention, whereas deqi of EA rather produced tingling and numbness.

It therefore seems advisable for EA treatments to first evoke deqi before applying electricity [92].

In line with this advice, Napadow et al. [55] also assumed that a brief manual stimulation which elicits sensation was more clinically relevant than longer duration (30 s–2 min) block-like stimulation.

Recently an fMRI study by Sun et al. [93] found different brain responses to various deqi sensations. They reported that BOLD responses around cortical areas were activation-dominated due to processing of somatosensory or pain signals. This study claimed a need for standardization of qualification and quantification methods of deqi [93].

In addition, Kong et al. [92] advised future researchers to use a standardized scale, for example, the MASS (Massachusetts general hospital acupuncture sensation scale), for documenting the elicited perception of deqi in order to make different deqi effects better comprehensible.

*(7) Placebo and Expectancy*. Acupuncture research protocols include a broad range of sham acupuncture models, ranging from placebo acupuncture which does not involve needling at all (like transcutaneous nerve stimulation), over nonpenetrating needling (like blunt overt needling on a patch or Streitberger needling), up to penetrating sham needling at a NMP or a NAP.

While Stux and Hammerschlag [94] suggested that all sham acupuncture models are convincing enough to patients to produce clinical equivalent effects, Birch et al. [95] stated that only placebo with needle insertion produces similar effects to those of acupuncture.

In agreement with the latter, Zaslawski et al. [80] declared that placebo without puncturing the skin could easily enable people to discriminate between sham and real acupuncture and might therefore not be effective.

However, no matter which kind of placebo is chosen, it is still hard to differentiate between the specific and nonspecific effects of sham acupuncture. As pain consists of a sensory and an affective component, both components also play an important role in the effects of placebo interventions [70].

In general, placebo analgesia is associated with striatal dopamine release [96], which points out a coupling of pain matrix and reward system. Similarly, Pariente et al. [22] found that the striatal reward system was activated when expectancy for (sham) acupuncture was high.

Studies by Huang et al. [66] and Kong et al. [97] corroborated this effect of expectancy. Huang et al. found that positive expectation increased acupuncture analgesia [66]. Kong et al. also reported that real and sham with high expectancy produced similar analgesia [97].

Due to so many similarities, Dhond et al. [5] investigated the differences between specific and placebo components of acupuncture. Real acupuncture, like placebo analgesia, modulated regions of pain perception in the limbic areas, sensorimotor and prefrontal cortices, brainstem nuclei, and cerebellum. While differences in the PFC and rACC indicated nonspecific pain expectancy, the modulation of amygdala, insula, and hypothalamus might suggest acupuncture specificity.

#### 5.2.2. Interpretation of Results

*(1) Interpretation of fMRI Results*. Activation and deactivation effects of certain brain areas in fMRI neuroimaging have to be interpreted with caution.

*(a) Activation and Deactivation*. Beissner and Henke [98] discussed the problem of activation and deactivation in their review on the difficulties of fMRI studies. They stated that deactivation could be regarded either as a reduction of neuronal activity compared to baseline due to acupuncture or as a neuronal activity stronger in the baseline than compared with acupuncture stimulation. Both possibilities cannot be distinguished, if compared to baseline.

*(b) Baseline*. This leads to the next problem of defining an adequate baseline. So far, most of the baselines tell people to close their eyes and think of nothing special or just to focus on the stimulation. However, Gusnard and Raichle [99] and Stark and Squire [100] stated that the cortical activity associated with this “low” baseline is neither well defined nor stationary.

Furthermore, Beckmann et al. [101] and DeLuca et al. [102] pointed out that brain activity does not cease when the subject is at rest, instead several resting state networks are active. Beissner and Henke [98] therefore proposed that a continuous attention task throughout the entire experiment would be a better option.

*(c) Interpretation of Spatial Effects*. One more difficulty of interpreting the broad range of fMRI signal changes in various brain areas was addressed by a review from Napadow et al. [103]. They pointed out that acupuncture is a complex somatosensory stimulus, as it elicits sensorimotor, affective, and higher cognitive/evaluative processes in the brain.

Therefore, they assumed that changes in certain brain areas could be specific for acupuncture analgesia, whereas others might be unspecific and rather linked with expectancy and emotional effects.

Their review data presented that the somatosensory cortex (S1, S2) could display early somatosensory stimuli, whereas the limbic brain regions (like hypothalamus, amygdala, ACC, and hippocampus) could mediate affective or emotional responses.

Similar to these findings, Pariente et al. [22] discussed the fact that many studies demonstrated modulation of the insula and the PFC. They presumed that the insula might play a specific role in pain processing whereas the PFC and its connections with the limbic system rather would to be linked with expectancy.

*(d) Measuring Temporal Effects*. It is supposed that, after needling stimulation in deep tissue with biochemical reactions to tissue damage, acupuncture still elicits prolonged effects after needle removal [104–107].

In line with this assumption, Napadow et al. [103] and Dhond et al. [107] also found that acupuncture does not just affect the brain during needling but can enhance the poststimulation spatial extent of resting brain networks.

Liu et al. [59] therefore pointed out that the common block- and event-related paradigms neither are suitable for long acupuncture stimulation periods similar to clinical treatment nor are they applicable for measuring the delayed and longer lasting effects of acupuncture. 

*(2) Interpretation of PET Results*. For PET studies the same precautions apply as discussed for fMRI.

*(3) Difficulties of Acupuncture EEG Studies*. Results of acupuncture EEG studies often report different and even contradicting effects. Li et al. [108] pointed out that these variations in EEG findings often depend on the physical and psychological state of the subject (e.g., attention). Hsu et al. [39] also noted that the participants' brain waves could be affected by environmental, mental, and physical factors such as anxiety and nervousness.

Some acupuncture EEG studies claimed that possible correlations with the ANS might as well modulate the EEG effects [109–111]. Another study also reported correlations between the EEG and autonomic functions in accordance with the acupuncture pain score [112].

On top of these inhomogeneous findings, EEG signals also often show a poor signal-to-noise ratio and reliable objectification can be difficult.

*(4) Difficulties of Acupuncture EP Studies*. For the interpretation of EPs in acupuncture trials, the possible influences of attentional manipulation and the activation of antinociceptive systems have to be taken into consideration.

Miltner et al. [113] earlier assumed that manipulation of a subject's attention could decrease the perception of pain. Shifting attention during acupuncture manipulation could therefore also influence the EP signals in acupuncture studies, especially those of long latency.

Kenntner-Mabiala [114] reinforced these early assumptions with a study proving that emotion and attention had distinct effects on EPs: modulation of N150 amplitudes reflected an affective pain modulation, whereas the modulation of P260 amplitudes was linked to attentional processes.

On top of these attention and pain modulating issues, Xu et al. [115] criticized that the conventional SEP methodology measuring fast conduction fibers might not be suitable for studying acupuncture mechanisms.

## 6. Conclusion

This review paper provides an overview of 33 neuroimaging studies, including trials with fMRI, PET, EEG, and EP. A majority of 25 studies compared MA versus sham acupuncture, while a minority of 7 trials compared EA versus sham, and only one study compared EA versus MA.

As acupuncture elicited a broad range of neurological responses, including somatosensory, affective, and cognitive aspects, only descriptive conclusions can be drawn. Evaluation of the very heterogeneous results shows evidence that in 29 out of 33 studies verum acupuncture elicits more/different modulation effects on neurological components measured by fMRI, PET, EEG, and EP than sham acupuncture.

For future studies using neuroimaging technology, more consistent methodology protocols are recommended. Stricter protocols would allow better comparison of results and could accelerate and consolidate advances in the field of acupuncture mechanism research.

In order to limit the most common mistakes while developing an acupuncture neuroimaging trial, it seems advisable for future researchers to seek advice from Witt et al.'s document “effectiveness guidance” [116] for clinical research. Furthermore, all researchers should closely follow the STRICTA guidelines in designing and reporting acupuncture studies.

## Figures and Tables

**Table 1:** fMRI studies on the effect of MA and EA.

Author	Year	Title	Objective	Outcomes
Bai et al. [44]	2010	Acupuncture modulates temporal neural responses in wide brain networks: evidence from fMRI study	Temporal investigation of (late) MA effects at ST36 (r) versus nearby NAP	They found that the amygdala and perigenual anterior cingulate cortex (pACC) exhibited increased activities during needling but decreased to reach a peak below the baseline. The PAG and hypothalamus presented intermittent activations across the whole session. Apart from the time-dependent responses, relatively persistent activities were also identified in the anterior insula and PFCs. In comparison, verum and sham shared a similar activation pattern in somatosensory areas (S1 and S2) during needling. However, during the postacupuncture resting period acupuncture at ST36 was followed by sustained activation of the S2, whereas acupuncture at NAP showed inhibition of the S1.

Cheng et al. [45]	2013	Exploration of whole brain networks modulated by acupuncture at analgesia acupoint ST36 using scale-specific wavelet correlation analysis	Investigation of MA effects at ST36 (r) versus nearby NAP effects on pairwise correlations between 90 cortical and subcortical regions	Their correlations presented frequency-specific modularity functional brain networks during poststimulus resting state following acupuncture at ST36 and NAP. Graph metrics in brain activity are different in verum and sham groups and also show that the brain network following manual acupuncture has higher global and local efficiency in parallel information transfer in the brain network compared with acupuncture at a NAP.

Cho et al. [46]	2010	fMRI study of effect on brain activity according to stimulation method at LI11, ST36: Painful pressure and acupuncture stimulation of same acupoints	Investigation of differences between MA versus painful sham stimulation at LI11 (l) versus ST36 (l)	In comparison to painful tactile stimulation, MA at LI11 led to activation of both sides of the parahippocampal gyrus, cerebellum, left side of thalamus, and right side of posterior cingulate regions. Acupuncture but not tactile stimulation at ST36 produced activation at the secondary motor cortex (M2), limbic system (cingulate gyrus, posterior cingulate), primary visual cortex, pons, and medulla regions, at the left BA6, BA8, and ACC. In comparison with the left LI11 acupuncture stimulation, left BA6, BA8, and ACC were more activated by the left ST36 acupuncture stimulation. Acupuncture activated more regions than painful tactile stimulation, especially areas of the limbic system, such as the parahippocampal gyrus and ACC.

Dong et al. [47]	2012	Tempo-spatial analysis of vision-related acupoint specificity in the occipital lobe using fMRI: An ICA study	Spatial and temporal investigation of the effects of MA at vision-related GB37 versus BL60 versus nearby NAP on the occipital lobe	Although the ICA of all kinds of acupuncture showed activity at the visual cortex V1 in the occipital lobe, temporal activities in this region differed for acupuncture at GB37 versus NAP, as well as for BL60 versus NAP.

Feng et al. [48]	2011	Investigation of the large-scale functional brain networks modulated by acupuncture	Spatial investigation of MA effects at ST36 (r) versus nearby NAP	Within a network of 90 predefined regions in the poststimulus resting brain, limbic/paralimbic regions (such as the amygdala, hippocampus, and anterior cingulate gyrus) emerged as network hubs after verum but not sham acupuncture. Compared with needling at a NAP, manual acupuncture at ST36 presented increased correlations, related with the limbic/paralimbic and subcortical regions (such as the insula, amygdala, and anterior cingulate gyrus) and thalamus. Decreased correlations for verum acupuncture were related with the sensory and frontal cortex.

Zyloney et al. [60]	2010	Manipulation of and sustained effects on the human brain induced by different modalities of acupuncture: An fMRI study	Spatial + temporal investigation of MA effects versus EA versus TEAS at ST36 (l)	Using a modified generalized linear model analysis to compare block-designed and resting-state fMRI scans they detected positive activation in the sensorimotor areas and negative activation in the default mode areas in both areas in both of the two 1-min-stimulation periods for tactile stimulation with a von Frey filament and in the first 1-min-stimulation of MA, EA, and TEAS. However, in the second 1-min-stimulation period, no positive activation result was observed and EA showed a more extensive deactivation compared to MA and TEAS.

Li et al. [49]	2010	Exploring vision-related acupuncture point specificity with multivoxel pattern analysis	Spatial investigation of MA effects at vision-related GB37 versus nearby NAP	They found different effects for verum acupuncture versus NAP in the subregions of occipital cortex (left cuneus of occipital gyrus and regions of lingual gyrus, middle occipital gyrus and fusiform gyrus), the limbic-cerebellar system (including insula, rACC and pACC, pons, amygdala, culmem in anterior lobe and declive of vermis in posterior lobe of cerebellum), and the somatosensory cortex. For GLM, the neutral response patterns of acupuncture stimulation at acupoints and NAP had multiple overlapping regions and did not differ significantly from each other.

Liu et al. [50]	2010	The hybrid GLM-ICA investigation on the neural mechanism of acupoint ST36: An fMRI study	Spatial and temporal investigation of MA effects at ST36 (r) versus nearby NAP	Their results showed manipulation-related effects and sustained acupuncture effects in the cortical-subcortical areas, including the ACC, ventrolateral prefrontal cortex (VLPFC), and supplementary motor area (SMA) and decreases in the S1 and S2. These reactions lasted until the resting period after needling, where then activations were induced in many regions including the insula, caudate, putamen, and thalamus.

Jiang et al. [58]	2013	Divergent neural processes specific to the acute and sustained phases of verum and sham acupuncture	Spatial investigation of immediate and delayed effects of MA at ST36 (r) versus nearby NAP	The immediate effect of verum as well as sham acupuncture consisted of signal changes in the limbic/paralimbic areas, neocortical regions, brainstem, and cerebellum. For a delayed effect, several regions showed strong functional connectivity. During the overall process of acupuncture, the insula played a critical role. Acupuncture at NAP produced positive activations with a small extent of spatial distribution and less intensive signal changes compared to ST36, mainly in the insula, S2, and cerebellum.

Liu et al. [51]	2012	Altered small-world efficiency of brain functional networks in acupuncture at ST36: A functional MRI study	Spatial investigation of MA effects at ST36 versus nearby NAP	The results presented increased local efficiency after acupuncture stimulation. No significant differences were found for sham acupuncture at a NAP. Significant effects of real acupuncture but not sham were detected on nodal degree of the left hippocampus. Point-related effects were observed in the ACC, frontal and occipital regions, while stimulation-related effects were found in various brain regions of frontal, parietal, and occipital cortex regions. Several limbic and subcortical brain regions exhibited point- and stimulation-related alterations in their regional homogeneity.

Liu et al. [52]	2012	Determining the precise cerebral response to acupuncture: An improved fMRI study	Investigation of effects of MA at LR3 versus nearby NAP, each tested with expectations versus no expectations	The superior part of the secondary visual cortex (V2) was activated in real acupuncture versus sham, and the interior part of V2 was activated in the other contrasting condition. All three contrasting conditions aimed to elicit cerebral responses to expectancy, the ipsilateral MFG, contralateral orbitofrontal cortex (OFC), contralateral S2, and contralateral cerebellum were activated. The contralateral DLPFC, temporal pole, and hippocampi uncus were activated in groups with expectation versus no expectation (medial frontal gyrus- and DLPFC-related expectancy is validated for emotion and cognitive control).

Liu et al. [53]	2013	Additional evidence for the sustained effect of acupuncture at the vision-related acupuncture point, GB37	Spatial and temporal investigation of MA effects at vision-related GB37 versus nearby NAP	GLM analysis showed a more extensive spatial distribution signal decrease in the limbic-cerebellar regions (such as the occipital cortex, pons, PH/Hipp, putamen, and cerebellum) but with a smaller signal increase (such as in the STG, S2, and thalamus). Special temporal investigation showed that the neural response evoked by acupuncture did not turn on and off rapidly but lasted longer, violating the basic assumption of standard GLM analysis. fMRI signals of the limbic-paralimbic-neocortical system increased, so that changes in the occipital cortex showed different temporal patterns between GB37 and NAP.

Murase et al. [54]	2013	Deconvolution analyses with tent functions reveal delayed and long-sustained increases of BOLD signals with acupuncture stimulation	Temporal + spatial investigation of MA effects versus von Frey filament sham acupuncture at LI4 (r) versus tactile stimulation right palm	MA showed activation on both sides in the S2 and the insula, on both sides in the S1, the primary motor cortex (M1), ACC, SMA, thalamus, and PFC. Sham acupuncture with von Frey filament showed that activation in the contralateral S1 and SMA and on both sides in the S2 and insula. Tactile stimulation showed activated areas in the contralateral S1, M1, and SMA and on both sides in the S2 and insula. Real acupuncture induced more widespread, more delayed, and long-sustained increases and decreases of BOLD signal in the somatosensory region and in areas related to pain perception.

Napadow et al. [55]	2013	Brain correlated of phasic autonomic response to acupuncture stimulation: An event-related fMRI study	Spatial + temporal investigation of ANS response and psychophysiological response patterns to MA at ST36 (l) versus SP9 (l) versus von Frey filament sham acupuncture at NAP (near ST36 (l))	GLM measurements showed that acupuncture events with strong skin conductance response produced greater anterior insula activation and acupuncture at SP9, which produced greater skin conductance response and also produced stronger sharp pain sensation and greater anterior insula activation. Acupuncture-induced heart rate (HR) deceleration was associated with greater DMN deactivation. This association was strongest for ST36, which produced more robust HR deceleration. DMN deactivation was significantly more pronounced across acupuncture stimuli producing HR deceleration versus those events characterized by acceleration.

Yeo et al. [56]	2010	Consecutive acupuncture stimulations lead to significantly decreased neural responses	Temporal investigation of repeated MA effects versus blunt sham acupuncture at BL62 (r)	They found that, after the first verum acupuncture stimulation block at the left BL62, the left hemisphere showed activation in the hypothalamus, thalamus, claustrum, cerebellum, inferior frontal gyrus, and the superior temporal gyrus, while the right hemisphere presented activation in the middle frontal gyrus. In both hemispheres, a significant focus of activation was found in the inferior parietal lobule. During the second block, only the cerebellum in the left hemisphere and the inferior parietal lobule in the right hemisphere were significantly activated, showing decreased activations during the second verum acupuncture stimulation. During sham, no significant brain activations were found.

You et al. [57]	2013	Altered hub configurations within default mode network following acupuncture at ST36: A multimodal investigation combining fMRI and MEG	Spatial + temporal investigation of MA effects at ST36 (r) versus nearby NAP on DMN hub configurations	They found that after sham acupuncture at NAP, the PCC remained to serve consistently as DMN hub across all five frequency bands. However, the PCC was regulated and only acted as a DMN hub within delta and gamma bands after verum acupuncture at ST36.

Liu et al. [59]	2011	Imaging the functional connectivity of the periaqueductal gray during genuine and sham electroacupuncture treatment	Spatial investigation of EA effects on PAG functional connectivity versus sham EA with Streitberger needles at LI3 (r) and LI4 (r), each with high versus low expectancy	They found greater connectivity between the PAG, left PCC, and precuneus in the comparison of verum EA versus Streitberger sham EA, whereas there was greater connectivity in the PAG and right anterior insula for sham EA. No significant differences were observed between high and low expectancy groups.

**Table 2:** PET studies on the effect of MA and EA.

Author	Year	Title	Objective	Outcomes
Biella et al. [24]	2001	Acupuncture produces central activations in pain regions	Investigation of cerebral blood flow (CBF) changes after MA at ST36 (bil) and LU5 (bil) versus two nearby NAPs (bil)	Verum acupuncture but not sham acupuncture activated the left anterior cingulus, the insulae bilaterally, the cerebellum bilaterally, the left superior frontal gyrus, and the right medial and inferior frontal gyri.

Dougherty et al. [35]	2008	A combined [11C] diprenorphine PET study and fMRI study of acupuncture analgesia	Investigation of changes in binding of opioid agonists and changes of heat pain after MA versus Streitberger sham acupuncture at LI4 (r)	In comparison to Streitberger acupuncture, they observed significant changes during verum acupuncture in the medial and lateral pain networks, such as opioid-binding decreases (associated with greater endogenous opioid release) in the right OFC, left medial PFC, right insula, and right thalamus, as well as binding increases in the bilateral insula, right medial PFC/ACC, left OFC, and right brainstem. An overlap of results between fMRI signals and [11C] diprenorphine blood pressure changes was only exhibited in the right medial OFC.

Hsieh et al. [17]	2001	Activation of the hypothalamus characterizes the acupuncture stimulation at the analgesic point in human: A positron emission tomography study	Investigation of point specific CBF changes during MA at LI4 (r) versus nearby NAP	In comparison to acupuncture at a NAP, only MA at LI4 elicited activation of the regional CBF (rCBF) in the areas of the hypothalamus with extension to midbrain, the insula, the ACC, and the cerebellum. In addition, a further comparison of needling with deqi contrasted with minimal manipulation acupuncture and showed activation in the hypothalamus and the cerebellum. The activation by deqi in the hypothalamus extended to the midbrain/brain stem when contrasted with the brain at rest. Minimal stimulation activated neither the hypothalamus nor the insula when compared with rest situation.

Lai et al. [36]	2009	A cerebral functional imaging study by positron emission tomography in healthy volunteers receiving true or sham acupuncture needling	Investigation of CBF changes during MA versus Streitberger needle versus overt blunt needling at TH5 (r)	For MA in comparison with overt blunt needling, more brain areas (BA7, 13, 18, 19, 21, 22, 27, 38, 40, 42, and 45) were activated, whereas, in comparison with Streitberger-like sham acupuncture, slightly less MA activation was found in the areas of BA13 and 42. During Streitberger-like sham acupuncture the areas BA4, 6, 7, 19, 22, and 41 showed activation.

Schlünzen et al. [37]	2007	Acupuncture of LI-4 in anesthetized healthy humans decreases cerebral blood flow in the putamen measured with positron emission tomography	Investigation of CBF changes during MA at LI4 (r) versus nearby NAP in anesthetized participants	Their results showed a decrease in CBF in the right medial frontal gyrus and in the left putamen for verum acupuncture. Acupuncture at a nearby NAP only caused a decrease of CBF in the right medial frontal gyrus.

**Table 3:** EEG studies on the effect of MA and EA.

Author	Year	Title	Objective	Outcomes
Cabrini et al. [38]	2006	Bispectral Index evaluation of the sedative effect of acupuncture in healthy volunteers	Evaluation of BIS changes due to bilateral MA at PC6, LR3, HT7, Yintang, ear point Shenmen versus nearby NAP	BIS values did not differ between true and sham acupuncture at any time point during the study period and BIS changes over time did not differ between the two treatments.

Hsu et al. [39]	2011	Variations of brain activities of acupuncture to TE5 of left hand in normal subjects	Evaluation of effects on the EEG during and after MA at TH5 (l) versus nearby NAP	During acupuncture stimulation, the theta energy was increased. During acupuncture, only alpha energy was noted to have statistical difference.

Kim et al. [40]	2008	The effect of acupuncture at PC-6 on the electroencephalogram and electrocardiogram	Evaluation of MA effects on the EEG during PC6 versus nearby NAP	EEG signals increased after acupuncture stimulation. In each frequency band, the average amplitude of EEG power was higher after acupuncture stimulation than after NAP stimulation.

Kim et al. [43]	2009	A characteristic estimation of bio-signals for electro-acupuncture stimulations in human subjects	Evaluation of bilateral EA effects at PC5 versus PC6 versus nearby NAP on the EEG	Their findings showed that during verum acupuncture the power spectrum of the low frequency bands in the EEG increased in all lobes.

Litscher [41]	2004	Effects of acupressure, manual acupuncture and laserneedle acupuncture on EEG bispectral index and spectral edge frequency in healthy volunteers	Evaluation of the effects on BIS during MA versus laser acupuncture versus acupressure at Yintang versus acupressure at NAP (near Yintang)	The study reports a decrease of BIS and spectral edge frequency values for acupressure and laser acupuncture at Yintang and for acupressure at the NAP, but not for manual acupuncture.

Streitberger et al. [42]	2008	Effects of verum acupuncture compared to placebo acupuncture on quantitative EEG and heart rate variability in healthy volunteers	Evaluation of the effects on the quantitative EEG during MA at LI4 (bil) versus Streitberger sham acupuncture at nearby NAP	In linear relation to heart rate variability (HRV) changes, verum acupuncture influenced the power EEG with increase in the alpha1-frequency of the occipital region with a shift of the alpha1/theta ratio to the benefit of alpha1 over all electrodes. A negative linear correlation was found between the theta-band of the quantitative EEG and the HRV parameters, and a negative linear correlation was also found between low frequency and alpha1 as well as between high frequency and alpha1.

**Table 4:** EP studies on the effect of MA and EA.

Author	Year	Title	Objective	Outcomes
Abad-Alegría and Pomarón [30]	2004	About the neurobiological foundations of the De-Qi-stimulus-response relation	Evaluation of EA effects without deqi during needle insertion at LI4 versus EA with deqi versus painful overstimulation versus EA at NAP on SEPs	Their measurements showed a direct relation between F-waves and SEPs with increasing electrostimulus, with main inflexion during deqi, whereas, with ongoing stimulation, greater variations took place, especially in case of SEP latency. In contrast, EA at a NAP did not produce any of the aforementioned effects.

Kvorning et al. [31]	2003	Acupuncture facilitates neuromuscular and oculomotor responses to skin incision with no influence on auditory evoked potentials under sevoflurane anaesthesia	Evaluation of bilateral EA effects at LI4, PC6, ST36, SP9, LR3, SP6 versus sham EA on AEPs	They found no significant difference of mid-latency or any other AEPs between the two groups, which could have correlated with the depth of anesthesia.

Meissner et al. [32]	2004	Acupuncture decreases somatosensory evoked + potential amplitudes to noxious stimuli in anesthetized volunteers	Evaluation of SEP changes after bilateral EA at ST36, SP6, LR3 versus sham EA	They detected a decrease in the magnitudes of late SEP amplitudes (P260) after verum but not sham EA.

Wei et al. [33]	2000	Early-latency somatosensory evoked potentials elicited by electrical acupuncture after needling acupoint LI-4	Evaluation of SEPs elicited by EA at LI4 (r) versus nearby NAP	Their results presented longer N1 and N2 latencies by acupuncture at LI4 as well as acupuncture at a nearby NAP than by median nerve stimulation, but showed no significant SEP differences between acupuncture at LI4 versus NAP.

Zeng et al. [34]	2006	Electroacupuncture modulates cortical activities evoked by noxious somatosensory stimulations in human	Temporal evaluation of EEG activities and evaluation of effects on painful SEPs after EA at LI4 (l) versus nearby NAP	EA at LI4 but not at a nearby NAP produced later-latency SEPs (P150) in bilateral ACC and attenuated pain specific amplitudes of P170 and N280 after median nerve stimulation.

**Table 5:** Results fMRI.

Author	Methodology				Needling details							Control intervention						Technology	
	Participants	Handedness	Groups	Sessions	Points (uni-/bilateral)	Needling depth	Manipulation	deqi	Retention time	Intervention 1	Group 1	Intervention 2	Group 2	Intervention 3	Group 3	Intervention 4	Group 4	Technical device	Software
Bai et al. [44]	16	Right	1	2	ST36 (r)	20–30 mm	90 sec, 1 Hz	yes	15 min	Manual	16	NAP	16					3 Tesla	SPM5
Cheng et al. [45]	32	Right	2	2	ST36 (r)	n/a	90 sec, 1 Hz	n/a	15 min	Manual	16	NAP	16					3 Tesla	SPM5
Cho et al. [46]	10	Right	1	4	LI11 (l), ST36 (l)	15–20 mm	3 × 30 sec, 2 Hz	yes	180 sec	Manual	10	Cotton tip	10					3 Tesla	SPM2
Dong et al. [47]	39	Right	3	1	GB37, BL60	n/a	2 × 30 sec, 1 Hz	yes	3 min 40 sec	Manual	13	NAP	13	BL60	13			3 Tesla	SPM5
Feng et al. [48]	14	Right	1	2	ST36 (r)	n/a	90 sec	yes	15 min	Manual	14	NAP	14					3 Tesla	SPM5
Zyloney et al. [60]	18	Right	1		ST36 (l)	15–25 mm	2 × 60 sec + 1 × 300 sec, 1-2 Hz	yes	10.5 min	Manual	18	vFrey	18	Electro	18	TEAS	18	3 Tesla	SPM5
Li et al. [49]	22	Right	2	1	GB37	n/a	2 × 30 sec	yes	3 min	Manual	11	NAP	11					3 Tesla	SPM5
Liu et al. [50]	18	Right	1	2	ST36 (r)	20–30 mm	90 sec, 1 Hz	yes	8.5 min	Manual	18	NAP	18					3 Tesla	SPM5
Jiang et al. [58]	14	n/a	1	2	ST36 (r)	10–20 mm	90 sec, 1 Hz	yes	15 min	Manual	14	NAP	14					3 Tesla	SPM5
Liu et al. [51]	18	Right	2	2	ST36	15 mm	3 × 60 sec, 2 Hz	Yes	20 min	Manual	9	NAP	9					1.5 Tesla	SPM5
Liu et al. [52]	41	Right	4	1	LR3	10 mm	120 sec, 1 Hz	Yes	2 min	Manual	11 + 10	NAP	10 + 9					1.5 Tesla	SPM2
Liu et al. [53]	22	Right	1	2	GB37	20–30 mm	2 × 60 sec, 1 Hz	Yes	220 sec	Manual	22	NAP	22					3 Tesla	SPM5
Murase et al. [54]	26	Right	2	1	LI4 (r)	15 mm	4 × 15 sec, 1 Hz	n/a	270 sec	Manual	13	vFrey	13					1.5 Tesla	SPM8
Napadow et al. [55]	18	Right	1	2	ST36 (l), SP9 (l)	20–30 mm	2 sec, 1 Hz	Yes	300 sec	Manual	18	vFrey	18					3 Tesla	FSL, AFMI
Yeo et al. [56]	15	Right	1	2	BL62 (r)	10 mm	2 × 30 sec, 2 Hz	Yes	4 min	Manual	15	Blunt needle	15					3 Tesla	SPM5
You et al. [57]	28	Right	2	1	ST36 (r)	15–25 mm	120 sec, 1 Hz	Yes	9 min	Manual	14	NAP	14					3 Tesla	SPM5
Liu et al. [59]	48	Right	4	1	LI3 (r), LI4 (r)	15 mm	2 Hz	Yes	25 min	Electro	n/a	Streitberger	n/a					3 Tesla	n/a

**Table 6:** Results PET.

Author	Methodology				Needling details							Control intervention				Technology		
	Participants	Handedness	Groups	Sessions	Points (uni-/bilateral)	Needling depth	Manipulation	deqi	Retention time	Intervention 1	Group 1	Intervention 2	Group 2	Intervention 3	Group 3	Imaging	Technical device	Software
Biella et al. [24]	13	n/a	1	2	ST36 (bil), LU5 (bil)	10–20 mm	n/a	Yes	25 min	Manual	13	2x NAP	13			PET H2(15)O bolus	GE-Advance	SPM96
Dougherty et al. [35]	12	Right	2	2	LI4 (r)	10 mm	3 × 420 sec, 3 Hz	Yes	29 min	Manual	6	Streitberger	6			PET [11C] diprenorphine	PC-4096	SPM2
Hsieh et al. [17]	16	Right	2	1	LI4 (r)	3 mm	30 sec, 2 Hz	Yes	180 sec	Manual	8	NAP	8			PET rCBF	n/a	SPM96
Lai et al. [36]	18	Right	3	1	TH5 (r)	15 ± 2 mm	1 Hz	Yes	19 min	Manual	9	Streitberger	9	Blunt needle	9	PET 18F-FDG	ECAT EXACT HR+	SPM2
Schlünzen et al. [37]	13	Right	2	1	LI4 (r)	10 mm	3 Hz	n/a	n/a	Manual + sevoflurane	7	NAP	6			PET CBF	ECAT EXACT HR	n/a

**Table 7:** Results EEG.

Author	Methodology				Needling details							Control intervention				Technology		
	Participants	Handedness	Groups	Sessions	Points (uni-/bilateral)	Needling depth	Manipulation	deqi	Retention time	Intervention 1	Group 1	Intervention 2	Group 2	Intervention 3	Group 3	Imaging	Technical device	Software
Cabrini et al. [38]	10	n/a	1	2	PC6, LR3, HT7, Yintang, ear Shenmen (bil)	n/a	n/a	Yes	20 min	Manual	10	NAP	20			BIS	n/a	n/a
Hsu et al. [39]	24	n/a	2	1	TH5 (l)	15 mm	n/a	Yes	20 min	Manual	12	NAP	12			EEG	Biopac brain wave detection helmets	IOPAC
Kim et al. [40]	10	n/a	1	3	PC6	5–10 mm	n/a	n/a	15 min	Manual	10	NAP	10			EEG	Biopac Systems	Daubechies
Kim et al. [43]	10	n/a	2	1	PC5 (bil) versus PC6 (bil)	0.3 mm	20 Hz	n/a	5 min	Electro	n/a	NAP	n/a			EEG	n/a	ADC
Litscher [41]	25	n/a	1	4	Yintang	5 mm	4 × 20 sec	n/a	10 min	Manual	25	NAP acupressure	25	Laser	25	EEG + BIS	Zipprep Electrodes, Aspect A-1000	n/a
Streitberger et al. [42]	20	n/a	1	2	LI4 (bil)	1 mm	15 sec	Yes	10 min	Manual	10	Streitberger at NAP	10			EEG	CATEEM	Vision Analyzer

**Table 8:** Results EP.

Author	Methodology				Needling details							Control intervention				Technology		
	Participants	Handedness	Groups	Sessions	Points (uni-/bilateral)	Needling depth	Manipulation	deqi	Retention time	Intervention 1	Group 1	Intervention 2	Group 2	Intervention 3	Group 3	Imaging	Technical device	Software
Abad-Alegría and Pomarón [30]	21	n/a	1	4	LI4	n/a	10 Hz	Yes	10 min	Electro + deqi	21	NAP	21	Electro + no deqi	21	SSEP	n/a	n/a
Kvorning et al. [31]	45	n/a	2	1	LI4, PC6, ST36, SP9, LR3, SP6 (bil)	5–15 mm	180 sec, 2 Hz + 80 Hz pulses	n/a	20 min	Electro	22	Sham electro	23			AEP	AEP monitor	ARX Aline
Meissner et al. [32]	16	n/a	2	1	ST36, SP6, LR3 (bil)	n/a	10 Hz	n/a	15 min	Electro	8	Sham electro	8			SEP	EEG	Vision Analyzer
Wei et al. [33]	11	Right	1	2	LI4 (r)	n/a	1 Hz	n/a	n/a	Electro	11	NAP	11			SEP	128-channel system	n/a
Zeng et al. [34]	24	Right	1	6	LI4 (l)	12.5 + 4.5 mm	2 Hz	n/a	n/a	Electro	24	NAP	24			SEP	64-channel Quikcaps Neuroscan ESI-128 system	SCAN 4.1

**Table 9:** Subgroup results table.

(A) Comparison of main interventions	
(a) MA versus EA	MA increased DMN connectivity and EA showed DMN deactivation [58]

(B) Verum versus sham	
EA versus sham	
(a) Streitberger needling	EA increased functional connectivity of PAG [60]
(b) Patch/tape	No difference of AEP after EA or sham [31]
	Decrease of late SEP amplitude after EA [32]
MA versus sham	
(a) Painful tactile stimulation	More areas activated by MA (ST36 > LI11) than painful stimulus [46]
(b) Blunt overt sham	More activation by (1st > 2nd) MA than blunt overt sham [56]
(c) Von Frey filament	More areas activated after MA than von Frey filaments + delayed, sustained in/decreases after MA [54]
	Stronger ANS responses (HR, skin conductance resistance) and DMN changes after ST36 and SP9 than sham [55]
(d) Streitberger needling	More areas with PET opioid agonist binding decrease after MA than Streitberger needle [35]
	MA influences qEEG power bands changes in linear relation with HRV changes [42]
Verum versus combined sham	
(a) EA	Correlation of SEP F-waves with increasing EA stimulation [30]
(b) MA	BIS decrease for acupressure, laser and pressure at NAP, not MA [41]
	More areas activated for MA in comparison with blunt and MA in comparison with Streitberger [36]

(C) Point specificity	
GB37 versus NAP	
MA	Different temporal activities for GB37, BL60 and NAP [47]
	ICA but not GLM showed more affected areas by GB37 than NAP [49]
	Wider spatial distribution, long-lasting responses for GB37 than NAP [53]
LI4 versus NAP	
(a) MA	More rCBF activation for LI4 (with deqi > without) than NAP [17]
	CBF decreases in more areas for LI4 than NAP [37]
(b) EA	Correlation of SEP F-waves with increasing EA stimulation [30]
	No difference in SEP for LI4 and NAP [33]
	LI4 but not NAP produced later latency SEP and attenuation of n. medianus amplitude [34]
ST36 versus NAP	
MA	Wider and sustained activation effects after ST36 than NAP [44]
	Higher network efficiency after ST36 than NAP [45]
	Different network correlations after ST36 and NAP [48]
	Manipulation-related and longer-lasting effects for ST36 than NAP [50]
	Immediate activation of larger areas and sustained, stronger functional connectivity for ST36 in comparison to NAP [59]
	Different nodal and point-related effects, but similar efficiency after ST36 and NAP [51]
	Changes of PCC action as DMN hub after ST36 but not NAP [57]

## References

[1] Liang F. R., Wu X. (2006). The developmental status and prospect of the science of acupuncture and moxibustion abroad. *Zhongguo Zhen Jiu*.

[2] Bodeker G., Packer L., Halliwell B., Ong C. N. (2005). Traditional & complementary medicine in health care: public policy dimensions. *Herbal Medicines: Molecular Basis of Biological Activity and Health*.

[3] Liu C. Z., Litscher G., Liang F. R., Kong J., Wang L. P. (2014). Deqi sensation in different kinds of acupuncture. *Evidence-Based Complementary and Alternative Medicine*.

[4] Linde K., Streng A., Jürgens S. (2005). Acupuncture for patients with migraine: a randomized controlled trial. *The Journal of the American Medical Association*.

[5] Dhond R. P., Kettner N., Napadow V. (2007). Neuroimaging acupuncture effects in the human brain. *Journal of Alternative and Complementary Medicine*.

[6] Logothetis N. K., Wandell B. A. (2004). Interpreting the BOLD signal. *Annual Review of Physiology*.

[7] Lindquist M. A. (2008). The statistical analysis of fMRI data. *Statistical Science*.

[8] Nilsson L. G., Markowitsch H. J. (1999). *Cognitive Neuroscience of Memory*.

[9] Telenczuk B., Baker S. N., Herz A. V. M., Curio G. (2011). High-frequency EEG covaries with spike burst patterns detected in cortical neurons. *Journal of Neurophysiology*.

[10] Kissin I. (2000). Depth of anesthesia and bispectral index monitoring. *Anesthesia and Analgesia*.

[11] Creutzfeldt O. D., Watanabe S., Lux H. D. (1966). Relations between EEG phenomena and potentials of single cortical cells. I. Evoked responses after thalamic and epicortical stimulation. *Electroencephalography and Clinical Neurophysiology*.

[12] Chen A. C. N., Richard Chapman C., Harkins S. W. (1979). Brain evoked potentials are functional correlates of induced pain in man. *Pain*.

[13] Miltner W. H. R., Weiss T. (1998). Brain electrical correlates of pain processing. *Zeitschrift für Rheumatologie*.

[14] Rainville P. (2002). Brain mechanisms of pain affect and pain modulation. *Current Opinion in Neurobiology*.

[15] Tracey I., Mantyh P. W. (2007). The cerebral signature for pain perception and its modulation. *Neuron*.

[16] Leung L. (2012). Neurophysiological basis of acupuncture-induced analgesia—an updated review. *Journal of Acupuncture and Meridian Studies*.

[17] Hsieh J.-C., Tu C.-H., Chen F.-P. (2001). Activation of the hypothalamus characterizes the acupuncture stimulation at the analgesic point in human: a positron emission tomography study. *Neuroscience Letters*.

[18] Wu M.-T., Hsieh J.-C., Xiong J. (1999). Central nervous pathway for acupunture stimulation: localization of processing with functional MR imaging of the brain—preliminary experience. *Radiology*.

[19] Hui K. K., Liu J., Makris N. (2000). Acupuncture modulates the limbic system and subcortical gray structures of the human brain: evidence from fMRI studies in normal subjects. *Human Brain Mapping*.

[20] Yoo S. S., Teh E. K., Blinder R. A., Jolesz F. A. (2004). Modulation of cerebellar activities by acupuncture stimulation: evidence from fMRI study. *NeuroImage*.

[21] Napadow V., Makris N., Liu J., Kettner N. W., Kwong K. K., Hui K. K. S. (2005). Effects of electroacupuncture versus manual acupuncture on the human brain as measured by fMRI. *Human Brain Mapping*.

[22] Pariente J., White P., Frackowiak R. S. J., Lewith G. (2005). Expectancy and belief modulate the neuronal substrates of pain treated by acupuncture. *NeuroImage*.

[23] Zald D. H. (2003). The human amygdala and the emotional evaluation of sensory stimuli. *Brain Research Reviews*.

[24] Biella G., Sotgiu M. L., Pellegata G., Paulesu E., Castiglioni I., Fazio F. (2001). Acupuncture produces central activations in pain regions. *NeuroImage*.

[25] Casey K. L. (1999). Forebrain mechanisms of nociception and pain: analysis through imaging. *Proceedings of the National Academy of Sciences of the United States of America*.

[26] Greicius M. D., Krasnow B., Reiss A. L., Menon V. (2003). Functional connectivity in the resting brain: a network analysis of the default mode hypothesis. *Proceedings of the National Academy of Sciences of the United States of America*.

[27] Mazoyer B., Zago L., Mellet E. (2001). Cortical networks for working memory and executive functions sustain the conscious resting state in man. *Brain Research Bulletin*.

[28] Shulman G. L., Fiez J. A., Corbetta M. (1997). Common blood flow changes across visual tasks: II. Decreases in cerebral cortex. *Journal of Cognitive Neuroscience*.

[29] Otti A., Noll-Hussong M. (2012). Acupuncture-induced pain relief and the human brain's default mode network—an extended view of central effects of acupuncture analgesia. *Forschende Komplementarmedizin*.

[30] Abad-Alegría F., Pomarón C. (2004). About the neurobiological foundations of the De-Qi—stimulus-response relation. *American Journal of Chinese Medicine*.

[31] Kvorning N., Christiansson C., Åkeson J. (2003). Acupuncture facilitates neuromuscular and oculomotor responses to skin incision with no influence on auditory evoked potentials under sevoflurane anaesthesia. *Acta Anaesthesiologica Scandinavica*.

[32] Meissner W., Weiss T., Trippe R. H., Hecht H., Krapp C., Miltner W. H. (2004). Acupuncture decreases somatosensory evoked potential amplitudes to noxious stimuli in anesthetized volunteers. *Anesthesia and Analgesia*.

[33] Wei H., Kong J., Zhuang D., Shang H., Yang X. (2000). Early-latency somatosensory evoked potentials elicited by electrical acupuncture after needling acupoint Ll-4. *Clinical EEG Electroencephalography*.

[34] Zeng Y., Liang X.-C., Dai J.-P. (2006). Electroacupuncture modulates cortical activities evoked by noxious somatosensory stimulations in human. *Brain Research*.

[35] Dougherty D. D., Kong J., Webb M., Bonab A. A., Fischman A. J., Gollub R. L. (2008). A combined [11C]diprenorphine PET study and fMRI study of acupuncture analgesia. *Behavioural Brain Research*.

[36] Lai X., Zhang G., Huang Y. (2009). A cerebral functional imaging study by positron emission tomography in healthy volunteers receiving true or sham acupuncture needling. *Neuroscience Letters*.

[37] Schlünzen L., Vafaee M. S., Cold G. E. (2007). Acupuncture of LI-4 in anesthetized healthy humans decreases cerebral blood flow in the putamen measured with positron emission tomography. *Anesthesia and Analgesia*.

[38] Cabrini L., Gioia L., Gemma M., Cedrati V., Crivellari M. (2006). Bispectral index evaluation of the sedative effect of acupuncture in healthy volunteers. *Journal of Clinical Monitoring and Computing*.

[39] Hsu S.-F., Chen C.-Y., Ke M.-D., Huang C.-H., Sun Y.-T., Lin J.-G. (2011). Variations of brain activities of acupuncture to TE5 of left hand in normal subjects. *The American Journal of Chinese Medicine*.

[40] Kim M. S., Kim H. D., Seo H. D., Sawada K., Ishida M. (2008). The effect of acupuncture at PC-6 on the electroencephalogram and electrocardiogram. *American Journal of Chinese Medicine*.

[41] Litscher G. (2004). Effects of acupressure, manual acupuncture and laserneedle acupuncture on EEG bispectral index and spectral edge frequency in healthy volunteers. *European Journal of Anaesthesiology*.

[42] Streitberger K., Steppan J., Maier C., Hill H., Backs J., Plaschke K. (2008). Effects of verum acupuncture compared to placebo acupuncture on quantitative EEG and heart rate variability in healthy volunteers. *Journal of Alternative and Complementary Medicine*.

[43] Kim M. S., Cho Y. C., Moon J. H., Pak S. C. (2009). A characteristic estimation of bio-signals for electro-acupuncture stimulations in human subjects. *The American Journal of Chinese Medicine*.

[44] Bai L., Tian J., Zhong C. (2010). Acupuncture modulates temporal neural responses in wide brain networks: evidence from fMRI study. *Molecular Pain*.

[45] Cheng H., Yan H., Bai L.-J., Wang B.-G. (2013). Exploration of whole brain networks modulated by acupuncture at analgesia acupoint ST36 using scale-specific wavelet correlation analysis. *Chinese Medical Journal*.

[46] Cho S.-Y., Jahng G.-H., Park S.-U., Jung W.-S., Moon S.-K., Park J.-M. (2010). FMRI study of effect on brain activity according to stimulation method at LI11, ST36: painful pressure and acupuncture stimulation of same acupoints. *Journal of Alternative and Complementary Medicine*.

[47] Dong M., Qin W., Sun J. (2012). Tempo-spatial analysis of vision-related acupoint specificity in the occipital lobe using fMRI: an ICA study. *Brain Research*.

[48] Feng Y., Bai L., Ren Y. (2011). Investigation of the large-scale functional brain networks modulated by acupuncture. *Magnetic Resonance Imaging*.

[49] Li L., Qin W., Bai L., Tian J. (2010). Exploring vision-related acupuncture point specificity with multivoxel pattern analysis. *Magnetic Resonance Imaging*.

[50] Liu P., Zhou G., Zhang Y. (2010). The hybrid GLM-ICA investigation on the neural mechanism of acupoint ST36: an fMRI study. *Neuroscience Letters*.

[51] Liu B., Chen J., Wang J. (2012). Altered small-world efficiency of brain functional networks in acupuncture at ST36: a functional MRI study. *PLoS ONE*.

[52] Liu H., Xu J., Shan B. (2012). Determining the precise cerebral response to acupuncture: an improved fMRI study. *PLoS ONE*.

[53] Liu J., Nan J., Xiong S., Li G., Qin W., Tian J. (2013). Additional evidence for the sustained effect of acupuncture at the vision-related acupuncture point, GB37. *Acupuncture in Medicine*.

[54] Murase T., Umeda M., Fukunaga M., Tanaka C., Higuchi A. T. (2013). Deconvolution analyses with tent functions reveal delayed and long-sustained increases of BOLD signals with acupuncture stimulation. *Magnetic Resonance in Medical Sciences*.

[55] Napadow V., Lee J., Kim J. (2013). Brain correlates of phasic autonomic response to acupuncture stimulation: an event-related fMRI study. *Human Brain Mapping*.

[56] Yeo S., Choe I.-H., van den Noort M., Bosch P., Lim S. (2010). Consecutive acupuncture stimulations lead to significantly decreased neural responses. *Journal of Alternative and Complementary Medicine*.

[57] You Y., Bai L., Dai R. (2013). Altered hub configurations within default mode network following acupuncture at ST36: a multimodal investigation combining fMRI and MEG. *PLoS ONE*.

[58] Jiang Y., Wang H., Liu Z. (2013). Manipulation of and sustained effects on the human brain induced by different modalities of acupuncture: an fMRI study. *PLoS ONE*.

[59] Liu J., Qin W., Guo Q. (2011). Divergent neural processes specific to the acute and sustained phases of verum and sham acupuncture. *Journal of Magnetic Resonance Imaging*.

[60] Zyloney C. E., Jensen K., Polich G. (2010). Imaging the functional connectivity of the periaqueductal gray during genuine and sham electroacupuncture treatment. *Molecular Pain*.

[61] Ulett G. A., Han S., Han J.-S. (1998). Electroacupuncture: mechanisms and clinical application. *Biological Psychiatry*.

[62] Schliessbach J., van der Klift E., Arendt-Nielsen L., Curatolo M., Streitberger K. (2011). The effect of brief electrical and manual acupuncture stimulation on mechanical experimental pain. *Pain Medicine*.

[63] Hsieh C.-L. (1998). Modulation of cerebral cortex in acupuncture stimulation: a study using sympathetic skin response and somatosensory evoked potentials. *The American Journal of Chinese Medicine*.

[64] Kong J., Li F., Li R. (2002). A pilot study of functional magnetic resonance imaging of the brain during manual and electroacupuncture stimulation of acupuncture point (LI-4 Hegu) in normal subjects reveals differential brain activation between methods. *Journal of Alternative and Complementary Medicine*.

[65] Li G., Cheung R. T. F., Ma Q.-Y., Yang E. S. (2003). Visual cortical activations on fMRI upon stimulation of the vision-implicated acupoints. *NeuroReport*.

[66] Huang W., Pach D., Napadow V. (2012). Characterizing acupuncture stimuli using brain imaging with fMRI—a systematic review and meta-analysis of the literature. *PLoS ONE*.

[67] Bäumler P. I., Simang M., Kramer S., Irnich D. (2012). Acupuncture point localization varies among acupuncturists. *Forschende Komplementarmedizin*.

[68] Choi E. M., Jiang F., Longhurst J. C. (2012). Point specificity in acupuncture. *Chinese Medicine*.

[69] Yang X.-G., Li Y., Tian X.-P., Liang F.-R. (2010). Comments on selection of non-acupoints beyond meridians in studies of acupuncture and moxibustion. *Journal of Traditional Chinese Medicine*.

[70] White A. R., Filshie J., Cummings T. M. (2001). Clinical trials of acupuncture: consensus recommendations for optimal treatment, sham controls and blinding. *Complementary Therapies in Medicine*.

[71] Chou P.-C., Chu H.-Y., Lin J.-G. (2011). Safe needling depth of acupuncture points. *Journal of Alternative and Complementary Medicine*.

[72] Ceccherelli F., Rigoni M. T., Gagliardi G., Ruzzante L. (2002). Comparison of superficial and deep acupuncture in the treatment of lumbar myofascial pain: a double-blind randomized controlled study. *Clinical Journal of Pain*.

[73] Park J. J., Akazawa M., Ahn J. (2011). Acupuncture sensation during ultrasound guided acupuncture needling. *Acupuncture in Medicine*.

[74] Zhang J.-H., Cao X.-D., Li J., Tang W.-J., Liu H.-Q., Feng X.-Y. (2007). Neuronal specificity of needling acupoints at same meridian: a control functional magnetic resonance imaging study with electroacupuncture. *Acupuncture & Electro-Therapeutics Research*.

[75] Han J.-S. (2003). Acupuncture: neuropeptide release produced by electrical stimulation of different frequencies. *Trends in Neurosciences*.

[76] Wong J. Y. (1999). *Manual of Neuro-Anatomical Acupuncture*.

[77] Sakai S., Hori E., Umeno K., Kitabayashi N., Ono T., Nishijo H. (2007). Specific acupuncture sensation correlates with EEGs and autonomic changes in human subjects. *Autonomic Neuroscience: Basic and Clinical*.

[78] Marcus P. (1994). Towards a dose of acupuncture. *Acupuncture in Medicine*.

[79] Li G., Jack C. R., Yang E. S. (2006). An fMRI study of somatosensory-implicated acupuncture points in stable somatosensory stroke patients. *Journal of Magnetic Resonance Imaging*.

[80] Zaslawski C. J., Cobbin D., Lidums E., Petocz P. (2003). The impact of site specificity and needle manipulation on changes to pain pressure threshold following manual acupuncture: a controlled study. *Complementary Therapies in Medicine*.

[81] Ho T.-J., Duann J.-R., Shen W.-C., Lin J.-G. (2007). Needling sensation: Explanation of incongruent conclusion drawn from acupuncture fMRI study. *Journal of Alternative and Complementary Medicine*.

[82] Ho T.-J., Duann J.-R., Chen C.-M. (2008). Carryover effects alter fMRI statistical analysis in an acupuncture study. *American Journal of Chinese Medicine*.

[83] Chen S., Guo S., Marmori F. (2013). Appraisal of the Deqi concept among contemporary Chinese acupuncturists. *Evidence-Based Complementary and Alternative Medicine*.

[84] He P. R. (1989). *Acupuncture Instrument and Acupuncture Therapy*.

[85] Cheng K., Yang J. S., Wang Y. Y. (2010). The opinion of academician X.N. Cheng of deqi: retaining needle and deqi. *China News of Traditional Chinese Medicine*.

[86] Peng J. S., Fei J. Z. (2008). *The Secret and Unique Skill of Acupuncture*.

[87] Han J. S., Terenius L. (1982). Neurochemical basis of acupuncture analgesia. *Annual Review of Pharmacology and Toxicology*.

[88] Hui K. K. K. S., Sporko T. N., Vangel M. G., Li M., Fang J., Lao L. (2011). Perception of Deqi by Chinese and American acupuncturists: a pilot survey. *Chinese Medicine*.

[89] Wang K. M., Yao S. M., Xian Y. L., Hou Z. L. (1985). A study on the receptive field of acupoints and the relationship between characteristics of needling sensation and groups of afferent fibres. *Scientia Sinica Series B*.

[90] Hendry S. H. C., Hsiao S. S., Bushnell M. C., Zigmond M. J., Bloom F. E., Landis S. C. (1999). Somatic sensation. *Fundamental Neuroscience*.

[91] Park J.-E., Ryu Y.-H., Liu Y. (2013). A literature review of de qi in clinical studies. *Acupuncture in Medicine*.

[92] Kong J., Gollub R., Huang T. (2007). Acupuncture De Qi, from qualitative history to quantitative measurement. *Journal of Alternative and Complementary Medicine*.

[93] Sun J., Zhu Y., Yang Y. (2013). What is the *de*-*qi*-related pattern of BOLD responses? A review of acupuncture studies in fMRI. *Evidence-Based Complementary and Alternative Medicine*.

[94] Stux G., Hammerschlag R. (2001). *Clinical Acupuncture: Scientific Basis*.

[95] Birch S., Hammerschlag R., Trinh K., Zaslawski C. (2002). The non-specific effects of acupuncture treatment: when and how to control for them. *Clinical Acupuncture and Oriental Medicine*.

[96] Scott D. J., Stohler C. S., Egnatuk C. M., Wang H., Koeppe R. A., Zubieta J.-K. (2007). Individual differences in reward responding explain placebo-induced expectations and effects. *Neuron*.

[97] Kong J., Kaptchuk T. J., Polich G. (2009). Expectancy and treatment interactions: a dissociation between acupuncture analgesia and expectancy evoked placebo analgesia. *NeuroImage*.

[98] Beissner F., Henke C. (2011). Methodological problems in fMRI studies on acupuncture: a critical review with special emphasis on visual and auditory cortex activations. *Evidence-Based Complementary and Alternative Medicine*.

[99] Gusnard D. A., Raichle M. E. (2001). Searching for a baseline: functional imaging and the resting human brain. *Nature Reviews Neuroscience*.

[100] Stark C. E. L., Squire L. R. (2001). When zero is not zero: the problem of ambiguous baseline conditions in fMRI. *Proceedings of the National Academy of Sciences of the United States of America*.

[101] Beckmann C. F., DeLuca M., Devlin J. T., Smith S. M. (2005). Investigations into resting-state connectivity using independent component analysis. *Philosophical Transactions of the Royal Society B: Biological Sciences*.

[102] DeLuca M., Beckmann C. F., de Stefano N., Matthews P. M., Smith S. M. (2006). fMRI resting state networks define distinct modes of long-distance interactions in the human brain. *NeuroImage*.

[103] Napadow V., Ahn A., Longhurst J. (2008). The status and future of acupuncture clinical research. *The Journal of Alternative and Complementary Medicine*.

[104] Zhao Z.-Q. (2008). Neural mechanism underlying acupuncture analgesia. *Progress in Neurobiology*.

[105] Bai L., Qin W., Tian J. (2009). Time-varied characteristics of acupuncture effects in fMRI studies. *Human Brain Mapping*.

[106] Bai L., Qin W., Tian J. (2009). Acupuncture modulates spontaneous activities in the anticorrelated resting brain networks. *Brain Research*.

[107] Dhond R. P., Yeh C., Park K., Kettner N., Napadow V. (2008). Acupuncture modulates resting state connectivity in default and sensorimotor brain networks. *Pain*.

[108] Li Z., Wang C., Mak A. F. T., Chow D. H. K. (2005). Effects of acupuncture on heart rate variability in normal subjects under fatigue and non-fatigue state. *European Journal of Applied Physiology*.

[109] Aftanas L., Golosheykin S. (2005). Impact of regular meditation practice on EEG activity at rest and during evoked negative emotions. *International Journal of Neuroscience*.

[110] Barry R. J., Rushby J. A. (2006). An orienting reflex perspective on anteriorisation of the P3 of the event-related potential. *Experimental Brain Research*.

[111] Jurysta F., Lanquart J.-P., Van De Borne P. (2006). The link between cardiac autonomic activity and sleep delta power is altered in men with sleep apnea-hypopnea syndrome. *American Journal of Physiology: Regulatory Integrative and Comparative Physiology*.

[112] Zhang W. T., Jin Z., Cui G. H. (2003). Relations between brain network activation and analgesic effect induced by low vs. high frequency electrical acupoint stimulation in different subjects: a functional magnetic resonance imaging study. *Brain Research*.

[113] Miltner W., Johnson R., Braun C., Larbig W. (1989). Somatosensory event-related potentials to painful and non-painful stimuli: effects of attention. *Pain*.

[114] Kenntner-Mabiala R. (2006). *Affective and attention-based modulation of somatosensory evoked potentials: the effects of emotion and attention on pain processing [Ph.D. thesis]*.

[115] Xu X., Shibasaki H., Shindo K. (1993). Effects of acupuncture on somatosensory evoked potentials: a review. *Journal of Clinical Neurophysiology*.

[116] Witt C. M., Aickin M., Cherkin D. (2014). Effectiveness guidance document (EGD) for Chinese medicine trials: a consensus document. *Trials*.

[117] Meyer-Lindenberg A. (2010). From maps to mechanisms through neuroimaging of schizophrenia. *Nature*.

